# Ceramide-transfer protein-mediated ceramide transfer is a structurally tunable flow-inducing mechanism with structural feed-forward loops

**DOI:** 10.1098/rsos.180494

**Published:** 2018-06-27

**Authors:** Giulia Giordano

**Affiliations:** Delft Center for Systems and Control, Delft University of Technology (TU Delft), 2628 CD Delft, The Netherlands

**Keywords:** dynamical systems, biological models, ceramide-transfer protein-mediated ceramidetransfer, trans-Golgi network, structural analysis, input–output influences

## Abstract

This paper considers two models of ceramide-transfer protein (CERT)-mediated ceramide transfer at the *trans*-Golgi network proposed in the literature, *short distance shuttle* and *neck swinging*, and seeks *structural* (parameter-free) features of the two models, which rely exclusively on the peculiar interaction network and not on specific parameter values. In particular, it is shown that both models can be seen as flow-inducing systems, where the flows between pairs of species are tuned by the concentrations of other species, and suitable external inputs can *structurally* regulate ceramide transfer. In the short distance shuttle model, the amount of transferred ceramide is *structurally* tuned by active protein kinase D (PKD), both directly and indirectly, in a coherent feed-forward loop motif. In the neck-swinging model, the amount of transferred ceramide is *structurally* tuned by active PI4KIIIβ, while active PKD has an ambivalent effect, due to the presence of an incoherent feed-forward loop motif that directly inhibits ceramide transfer and indirectly promotes it; the *structural* role of active PKD is to favour CERT mobility in the cytosol. It is also shown that the influences among key variables often have *structurally* determined steady-state signs, which can help falsify the models against experimental traces.

## 1. Introduction

Located next to the endoplasmatic reticulum (ER) and close to the cell nucleus, the Golgi apparatus [1,2] has a fundamental role in the life of most eukaryotic cells [3–5]: proteins and lipids synthesized at the ER reach the Golgi apparatus, where they are sorted, appropriately modified, labelled for delivery to a specific location and then sent to their target destination. The Golgi apparatus consists of a stack of several cisternae, delimited by lipid membranes, grouped as *cis*-cisternae (the closest to the nucleus), medial-cisternae (in the central part) and *trans*-cisternae (the farthest from the nucleus). The outermost cisternae in the *cis* and *trans* faces, respectively, constitute the *cis*-Golgi network (CGN) and the *trans*-Golgi network (TGN), and handle proteins and lipids that are, respectively, received and released by the apparatus. When a molecule reaches the TGN, it is typically packaged into a vesicle [6,7] that carries it to its destination. However, a molecule can also leave the TGN due to *non-vesicular lipid transport* mechanisms [8–10], which do not involve vesicle formation. This intracellular lipid transport occurs at the membrane contact sites (MCSs) between the ER membrane and various organelles [11, table 1]: at the MCSs, the external lipid double layers of the two organelles are sufficiently close to allow for the exchange of membrane lipids, which is greatly facilitated by lipid-transfer proteins (LTPs). At the MCSs between the ER and the TGN, the ceramide-transfer protein (CERT) is highly specific for the transport of ceramide (a sphingolipid) [11–15]. In mammalian cells, ceramide is synthesized at the ER and a CERT-mediated transport process brings it to the TGN, where sphingomyelin synthase (SMS) converts it into sphingomyelin: SMS catalyses the conversion of ceramide and phosphatidylcholine (PC) into sphingomyelin (SM) and diacylglycerol (DAG) [16–20].

**Figure 1.**
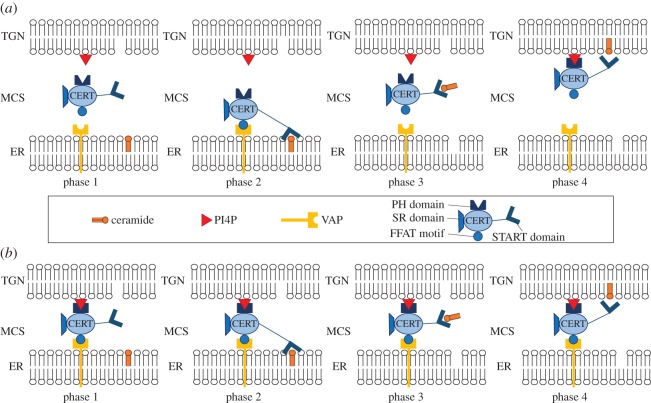
Models for CERT-mediated ceramide transfer at the ER–TGN membrane contact sites: phases of the transfer process in the *short distance shuttle* model (*a*) and the *neck swinging* model (*b*). Short distance shuttle: CERT is completely unbound and free to move between the two membranes (phase 1); CERT binds to the ER (because its FFAT motif binds to the VAP molecules present in the ER membrane [12,13,22,25,26]) and its START domain binds to ceramide and removes it from the ER (phase 2); CERT is detached from the ER and moves towards the TGN (phase 3); CERT binds to the TGN (because its PH domain binds to the PI4P molecules present on the TGN membrane [13]) and releases ceramide in the TGN (phase 4); the transfer cycle restarts when CERT detaches from the TGN and is again free to move (phase 1). Neck swinging: to transport ceramide, CERT needs to be bound to both the ER and the TGN membranes (phase 1), so that its START domain can bind to ceramide at the ER (phase 2), remove ceramide from the ER and bend towards the TGN (phase 3), and finally release ceramide in the TGN (phase 4).

CERT-mediated non-vesicular transport of ceramide at the ER–TGN MCSs relies on a complex network of regulatory interactions [14,21], whose exact mechanism is still unclear [17,22–24]. Two different models have been proposed: ‘short distance shuttle’ [12,24] and ‘neck swinging’ [3,14,24]. In the short distance shuttle model, the trafficking of ceramide occurs because CERT moves through the cytosol, continuously shuttling between the ER and the TGN membranes [12] (figure 1*a*). In the neck-swinging model, CERT can extract ceramide from the ER membrane and release it at the TGN membrane only when it is simultaneously bound to both membranes [3,14] (figure 1*b*). This paper studies the mathematical description first proposed [27] for these two models, which captures the interplay of PKD, PI4KIIIβ and CERT, the key proteins involved in the mechanism, on an average cellular level in mammalian cells. In Weber *et al.* [27], the two quantitative dynamical models are calibrated by estimating the parameters from experimental data based on Bayesian inference methods, and then validated against experimental data.

Here, the two models are analysed and compared with mathematical tools that help better understand the design principles behind CERT-mediated ceramide transfer.

Improving the understanding of this transport phenomenon is of high interest, not only in itself, but also for therapeutic applications. Identifying how to regulate the ER–TGN ceramide transfer (and thus SM synthesis) is fundamental to *controlling* several related processes and allowing for novel therapies: targeted biotechnological interventions on the TGN regulatory system could, for instance, optimize the secretion rate of therapeutic proteins in mammalian cells [27,28]. As an excessive conversion of ceramide correlates with resistance of cancer cells to drugs and chemotherapy [29], promising anti-oncogenic therapeutic approaches rely on the ability of acting on (and blocking) CERT-mediated ceramide transfer [30]; on the other hand, a lack of CERT can induce cellular dysfunctions [31]. Therefore, to design suitable interventions, the complex interplay of lipids and proteins at the TGN needs to be carefully understood.

Predictive mathematical models are crucial to revealing essential features of biological systems [32]: dynamical systems that describe the functioning of cells and pathways can be analysed with system-theoretic and control-theoretic methods [33–35] to get more insight into the mechanisms of life and assess key properties and behaviours. However, models are inherently plagued by uncertainty, due to the lack of exact quantitative knowledge and the variability of parameters present in nature; nevertheless, biological systems are incredibly robust [36,37] and able to perform their task in the most different conditions [38–40]. Therefore, it is often necessary—and extremely interesting—to assess the behaviour of a biological system based just on limited, qualitative information. A *structural (parameter-free) analysis* is then performed to find *structural properties* [32,41–44] that hold regardless of parameter values (within a feasible domain) and exclusively rely on how the key players are interconnected. Structural approaches have been developed to assess whether, *for any feasible choice of the parameter values*, a biological system enjoys a fundamental property or preserves a fundamental qualitative behaviour (such as, for instance, stability [45–47], oscillations or multistability [48–52], signed input–output influences [53]).

This paper adopts a structural viewpoint to highlight the fundamental differences between the short distance shuttle model and the neck-swinging model of CERT-mediated ceramide transfer, and reveals structural features of the two models that *do not depend on parameter values* but are rooted in the networked *structure* of the interactions among key molecules. To this aim, the models are cast into the mathematical framework of flow-inducing networks [54] and a structural algorithm [53] is employed to understand how variations in a variable can modify the steady-state value of other variables, independent of the value of the system parameters.

## 2. Models and methods

### 2.1. The models

CERT-mediated non-vesicular ceramide transfer involves the following essential lipids and proteins: the sphingolipid ceramide, which has to be transferred from ER to TGN; the glycerolipid DAG, produced at the TGN [18] and able to indirectly activate the protein PKD [55,56]; the glycerolipid phosphatidylinositol (PI), along with its phosphorylated version phosphatidylinositol 4 phosphate (PI4P), that attracts CERT to the TGN by binding to its PH domain [13] and is thus essential for ceramide transport [57]; protein kinase D (PKD), existing in several isoforms [58,59], two of which, when activated by DAG, are able to phosphorylate PI4KIIIβ and CERT at the TGN [21,55,56,60]; the cytoplasmic lipid kinase phosphatidylinositol-4-kinase-IIIβ (PI4KIIIβ), a protein that, when activated through phosphorylation by PKD, triggers conversion of PI into PI4P, hence attracting CERT to the TGN [61–63]; the cytoplasmic lipid-transfer protein CERT, highly specific for ceramide transfer [11], endowed with multiple functional domains for binding to the ER and the TGN membranes to extract and release ceramide, and with phosphorylation sites that allow the transfer process to be tuned. The PH domain of CERT is associated with binding to the TGN [3,64,65], the FFAT motif with binding to the ER [14,26], the START domain with binding to ceramide [3,66–68], and the SR motif with the regulation of binding affinities [25,60,69].

The considered ordinary differential equation (ODE) models [27] take into account the different possible states of the key molecules PKD, PI4KIIIβ and CERT and the fundamental interactions among them, which are now briefly summarized.

**Figure 2.**
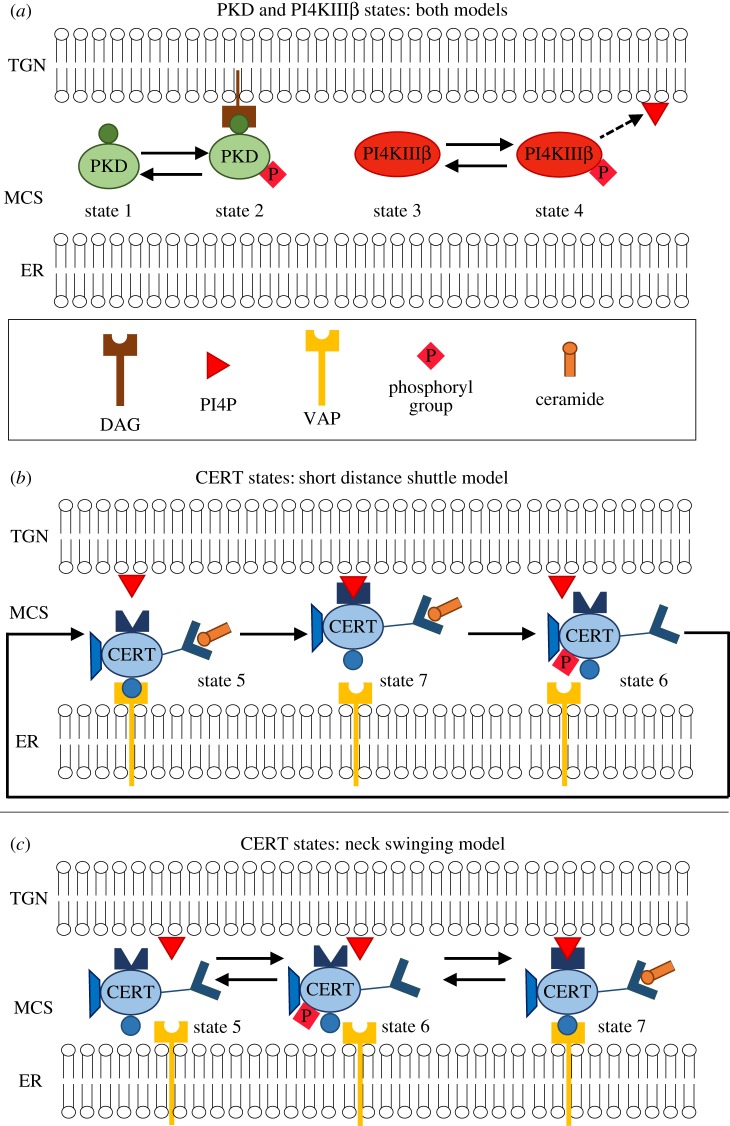
Key molecules at the interface between the ER and the TGN can be in different states. (*a*) Inactive PKD (state 1) and active PKDpDAG (state 2); inactive PI4KIIIβ (state 3) and active PI4KIIIβp (state 4). (*b*) In the *short distance shuttle* model, CERT can be either bound to ER (state 5), bound to TGN (state 7) or unbound and phosphorylated (state 6). (*c*) In the *neck-swinging* model, CERT can be either unbound and unphosphorylated (state 5), unbound and phosphorylated (state 6) or bound to both the ER and the TGN (state 7).

As shown in figure 2*a*, both PKD and PI4KIIIβ can be present in two forms: PKD, inactive and unphosphorylated (state 1) and PKDpDAG, active and phosphorylated, bound to DAG at the TGN (state 2); PI4KIIIβ, inactive and unphosphorylated (state 3) and PI4KIIIβp, active and phosphorylated (state 4). The lipid PI4P, which recruits CERT to the TGN, is produced by PI4KIIIβp. PKDpDAG, by direct phosphorylation, can either activate PI4KIIIβ or induce the detachment of CERT from the TGN membrane. Also, PKD can be activated by the CERT-mediated ceramide transfer: at the TGN, ceramide and PC are converted into SM and DAG due to SMS [16,18–20], and the increased quantity of DAG promotes the recruitment of PKD at the TGN, hence its activation.

In the short distance shuttle model (figure 2*b*), CERT can be present in three forms: CERTaER, bound to the ER and unphosphorylated (state 5); CERTp, unbound and phosphorylated (state 6); CERTaTGN, bound to the TGN and unphosphorylated (state 7). CERT is able to deliver a ceramide molecule only when it is bound to the TGN (state 7). After CERT has delivered ceramide to the TGN, it needs to detach from the TGN (via PKD-induced phosphorylation, which converts it to state 6) and then to be dephosphorylated and bound to the ER membrane (state 5) so as to be able to pick up another ceramide molecule at the ER. Then, CERT can detach from the ER and bind again to the TGN (state 7), due to the action of PI4KIIIβp, to deliver the ceramide molecule.

In the neck-swinging model (figure 2*c*), CERT can be present in three forms: CERTa, unbound and unphosphorylated (state 5); CERTp, unbound and phosphorylated (state 6); CERTaERTGN, bound to both membranes and unphosphorylated (state 7). CERT is only able to transfer ceramide from the ER to the TGN when it is in the double-bound unphosphorylated state 7. PKD-induced phosphorylation detaches CERT from both the TGN and ER membranes (bringing it from state 7 to state 6) and thus interrupts the transfer process. Then, spontaneous dephosphorylation can bring CERT to state 5, while spontaneous phosphorylation brings it back to state 6. The transition from state 6 to state 7 is mediated by the action of PI4KIIIβp, which attracts CERT to the TGN (concurrently, dephosphorylation and binding to the ER also occur), and is required to reactivate ceramide transfer.

**Figure 3.**
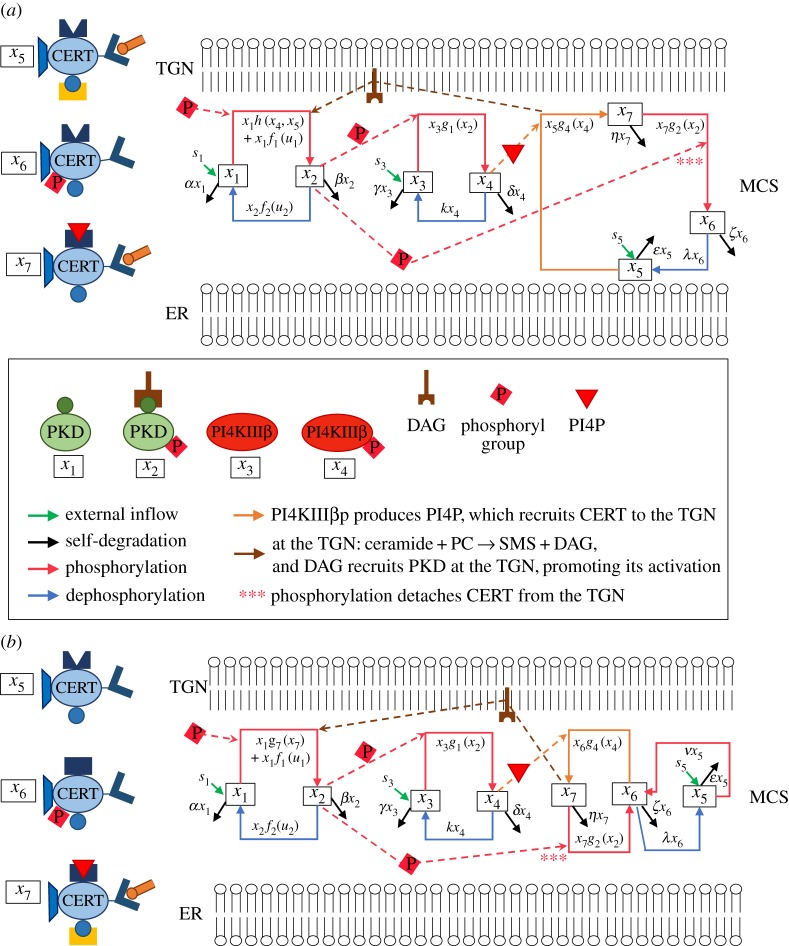
Reaction diagrams for the short distance shuttle model (2.1) (*a*) and the neck-swinging model (2.2) (*b*): *x*_1_ is associated with PKD (state 1), *x*_2_ with PKDpDAG (state 2), *x*_3_ with PI4KIIIβ (state 3), *x*_4_ with PI4KIIIβp (state 4); in the short distance shuttle model, *x*_5_ with CERTaER (state 5), *x*_6_ with CERTp (state 6) and *x*_7_ with CERTaTGN (state 7); in the neck-swinging model, *x*_5_ with CERTa (state 5), *x*_6_ with CERTp (state 6) and *x*_7_ with CERTaERTGN (state 7). In both models: an external inflow produces *x*_1_, *x*_3_ and *x*_5_; all species self-degrade; *x*_1_ phosphorylates into *x*_2_ [at rate *x*_1_*f*_1_(*u*_1_)]; *x*_2_ dephosphorylates into *x*_1_ [*x*_2_*f*_2_(*u*_2_)]; *x*_4_ dephosphorylates into *x*_3_ [*κx*_4_]; *x*_3_ phosphorylates into *x*_4_ due to *x*_2_ [*x*_3_*g*_1_(*x*_2_)]; *x*_7_ phosphorylates into *x*_6_ due to *x*_2_ [*x*_7_*g*_2_(*x*_2_)]; *x*_6_ dephosphorylates into *x*_5_ [λ*x*_6_]. In the short distance shuttle model: *x*_4_-produced PI4P converts *x*_5_ into *x*_7_ [*x*_5_*g*_4_(*x*_4_)]; *x*_1_ turns into *x*_2_ due to DAG generated by ceramide transfer, which depends on both ceramide-carrying CERT at the ER and on PI4KIIIβp that recruits it at the TGN [*x*_1_*h*(*x*_4_, *x*_5_)]. In the neck-swinging model: *x*_4_-produced PI4P converts *x*_6_ into *x*_7_ [*x*_5_*g*_4_(*x*_4_)]; *x*_5_ phosphorylates into *x*_6_ [*ϵx*_5_]; *x*_1_ turns into *x*_2_ due to DAG generated by ceramide transfer, which depends on double-bound CERT [*x*_1_*g*_7_(*x*_7_)].

The overall process, with the reactions occurring in the two models according to Weber *et al.* [27], is visualized in the reaction diagrams in figure 3. In the ODE models, the state variables correspond to the concentrations of the species discussed above: *x*_*i*_ is the concentration of the molecule in state *i*. The dots indicate time derivatives. Then, the short distance shuttle system is

2.1 $$\begin{array}{rl} {\dot{x}}_{1} & =-x_{1}h(x_{4},x_{5})-x_{1}f_{1}(u_{1})+x_{2}f_{2}(u_{2})+s_{1}-\alpha x_{1},\\ {\dot{x}}_{2} & =x_{1}h(x_{4},x_{5})+x_{1}f_{1}(u_{1})-x_{2}f_{2}(u_{2})-\beta x_{2},\\ {\dot{x}}_{3} & =\kappa x_{4}-x_{3}g_{1}(x_{2})+s_{3}-\gamma x_{3},\\ {\dot{x}}_{4} & =-\kappa x_{4}+x_{3}g_{1}(x_{2})-\delta x_{4},\\ {\dot{x}}_{5} & =\lambda x_{6}-x_{5}g_{4}(x_{4})+s_{5}-\varepsilon x_{5},\\ {\dot{x}}_{6} & =-\lambda x_{6}+x_{7}g_{2}(x_{2})-\zeta x_{6}\\ {\dot{x}}_{7} & =x_{5}g_{4}(x_{4})-x_{7}g_{2}(x_{2})-\eta x_{7}, \end{array}\}$$

while the neck-swinging system is

2.2 $$\begin{array}{rl} {\dot{x}}_{1} & =-x_{1}g_{7}(x_{7})-x_{1}f_{1}(u_{1})+x_{2}f_{2}(u_{2})+s_{1}-\alpha x_{1},\\ {\dot{x}}_{2} & =x_{1}g_{7}(x_{7})+x_{1}f_{1}(u_{1})-x_{2}f_{2}(u_{2})-\beta x_{2},\\ {\dot{x}}_{3} & =\kappa x_{4}-x_{3}g_{1}(x_{2})+s_{3}-\gamma x_{3},\\ {\dot{x}}_{4} & =-\kappa x_{4}+x_{3}g_{1}(x_{2})-\delta x_{4},\\ {\dot{x}}_{5} & =-\nu x_{5}+\lambda x_{6}+s_{5}-\varepsilon x_{5},\\ {\dot{x}}_{6} & =\nu x_{5}-\lambda x_{6}-x_{6}g_{4}(x_{4})+x_{7}g_{2}(x_{2})-\zeta x_{6}\\ {\dot{x}}_{7} & =x_{6}g_{4}(x_{4})-x_{7}g_{2}(x_{2})-\eta x_{7}. \end{array}\}$$

The positive constants *s*_1_, *s*_3_ and *s*_5_ represent external inflows, while the controlled input signals *u*_1_ and *u*_2_ are naturally zero and can be artificially set to positive values; all the coefficients represented by Greek letters are positive.

The quantitative kinetic model built in Weber *et al.* [27] is based on chemical reaction kinetics, and resorts to linear mass action kinetics for degradation rates, activation of PKD and dephosphorylation of CERT at the ER, and to enzymatic kinetics of the Michaelis–Menten type for regulatory effects across key proteins. In particular, the functions *f*_*i*_, *g*_*i*_ and *h* are chosen [27] as *f*_*i*_(*z*) = *a*_*i*_(1 + *b*_*i*_*z*), *g*_*i*_(*z*) = *a*_*i*_(*z*/(*z* + *b*_*i*_)), *h*(*z*, *y*) = *g*_*j*_[*yg*_*i*_(*z*)] = *a*_*j*_(*ya*_*i*_(*z*/(*z* + *b*_*i*_))/(*b*_*j*_ + *ya*_*i*_(*z*/(*z* + *b*_*i*_)))), where *a*_*i*_, *b*_*i*_, *a*_*j*_, *b*_*j*_ > 0; with this specific choice of the functions, the parameter values are estimated from experimental data, and the resulting models are validated by comparing simulation results with experimental traces.

This paper, conversely, considers a generalization of the models in Weber *et al.* [27], where now *f*_*i*_( · ), *g*_*i*_( · ) and *h*( · , · ) are *generic functions* that satisfy the following assumption.

**Assumption 2.1.**

Functions *f*_*i*_( · ), *g*_*i*_( · ) and *h*( · , · ) are non-negative and differentiable, with positive partial derivatives in the positive orthant. In particular, *f*_*i*_( · ) are strictly positive, also when their argument is zero: *f*_*i*_(0) > 0.

This generalization is motivated by the goal of studying and comparing the two models with a *structural* approach: in the following, a *structural* mathematical analysis is performed for the two generalized models, which does not rely on specific function or parameter choices and provides *parameter-free* insight into the biological functioning. Therefore, the results derived in this paper hold for *any* choice of the positive parameter values and of the model functions *f*_*i*_( · ), *g*_*i*_( · ) and *h*( · , · ) satisfying assumption 2.1.

The functional expressions and the parameter values of the original models in Weber *et al.* [27] are still used in this paper for the numerical simulations reported in §3.5, where the model parameters and the initial conditions used for the simulations are specified. However, *no information about the model initial conditions, parameter values and functional expressions is needed for the following theoretical analysis*, because the aim is indeed to give results that are completely independent of uncertain model parameters and of the specific choice of the functions (as long as they satisfy assumption 2.1), as well as of the initial conditions: the results always hold if the system *structure* is preserved. This ensures *robustness of the results* with respect to *all structure-preserving modelling choices*.

### 2.2. The framework: flow-inducing networks

Flow-inducing networks are a general class of models introduced in Giordano & Blanchini [54], which are well suited to describe the behaviour of many biochemical systems where a species can boost a reaction without being affected by it (to a first approximation). In mathematical terms, a flow-inducing network is a dynamical system resulting from the interconnection of various dynamical subsystems, or modules. Each module is compartmental, and thus groups positive state variables subject to mass conservation: the sum of the variables in the module is constant, at all time instants. The time evolution of the variables within each module is due to flows among those variables, which can be either spontaneous (proper flows), or tuned by the value of variables belonging to other modules (flows tuned by flow-inducing signals). A minimal example of flow-inducing network is

2.3 $$\begin{array}{rl} e & =\text{incoming signal},\\ \dot{a} & =-f_{a}(a,e)+f_{b}(b)\\ \dot{b} & =+f_{a}(a,e)-f_{b}(b), \end{array}\}$$

where *a* (respectively, *b*, *e*) is the concentration of the biochemical species *A* (respectively, *B*, *E*). There is a module including *A* and *B*, where the sum of the concentrations is constant: $\dot{a}+\dot{b}=0$. Species *E* is part of a different module, evolves according to its own dynamics and can influence the *A*–*B* module. The individual concentrations of *A* and *B* evolve over time due to: the flow from *B* to *A*, which is spontaneous, because the rate of conversion of *B* into *A*, *f*_*b*_(*b*), depends on the concentration of *B* only; and the flow from *A* to *B*, which is tuned by species *E* through a *flow-inducing signal*. In fact, the rate of conversion of *A* into *B*, *f*_*a*_(*a*, *e*), depends not only on the concentration of *A* but also on the concentration of *E*, a species that belongs to another module.

**Figure 4.**
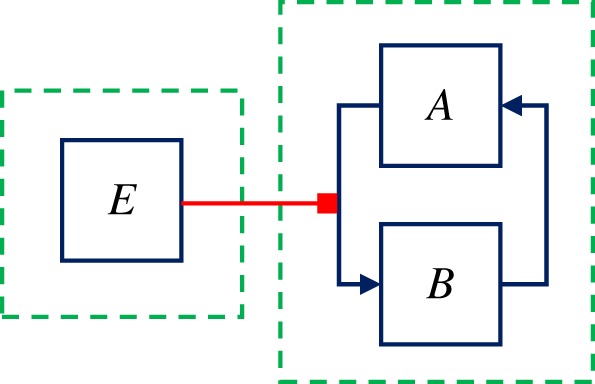
Graph representation of a minimal flow-inducing network, represented by the system (2.3): the state variables are represented by nodes (blue squares), and variables belonging to the same module are grouped by dotted green boxes; the flows among variables within the same module are represented by arcs (blue pointed arrows) that connect the nodes; the flow-inducing signals are represented by meta-arcs (red hammer head arrow) that connect a node in a module to an arc in a different module.

A flow-inducing network can be associated with a *graph*: the graph representing system (2.3) is in figure 4. In the graph representation, the state variables (concentrations of chemical species) are associated with nodes (the blue squares), the flows with arcs (blue pointed arrows) that connect the nodes, and the flow-inducing signals with meta-arcs (red hammer head arrows) that connect a node to an arc. Nodes associated with variables in the same module are grouped by dotted green boxes. Therefore, the blue arcs always connect nodes within a module, inside the dotted green box, while the red meta-arcs always connect a node in a module to an arc in a different module.

In the models (2.1) and (2.2), there is no mass conservation for the total species. The two considered models of CERT-mediated ceramide transfer exactly fit into the mathematical framework of flow-inducing networks, as shown in Giordano & Blanchini [54], if an external production rate exactly compensates self-degradation for all variables, thus ensuring mass conservation in each of the modules. However, an analysis in the flow-inducing network framework can be performed for the original systems (2.1) and (2.2) even though there is no mass conservation, because this does not alter the core flow-inducing structure of the models (this aspect is discussed in more detail in the electronic supplementary material, §3).

**Figure 5.**
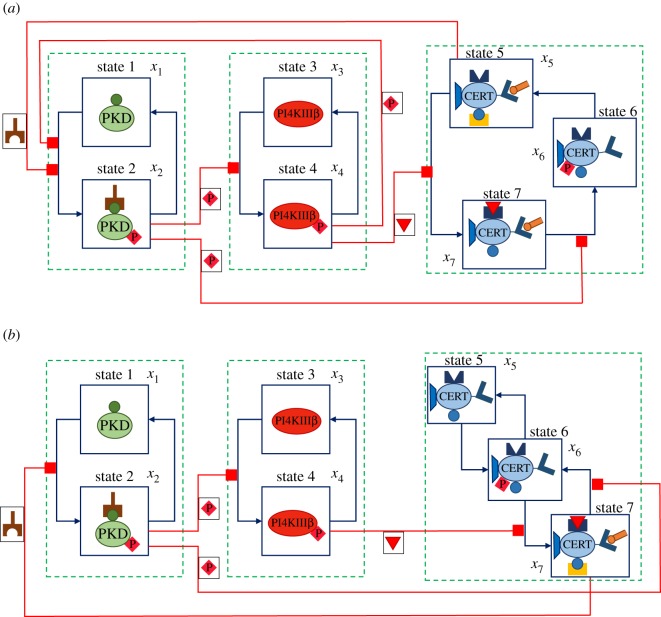
Model graphs as flow-inducing networks: (*a*) short distance shuttle and (*b*) neck swinging. The main differences between the models are highlighted. In the short distance shuttle model, the CERT-subsystem has a circular structure, because extraction and delivery of ceramide requires the temporary detachment of CERT from the TGN, and both PKDp (state 2) and PI4KIIIβp (state 4) favour this circulation; in the neck-swinging model, it has a cascaded double-switch structure, where the transfer process, related to the amount of CERT in state 7, can be switched off by PKDp (state 2) and switched on by PI4KIIIβp (state 4). Also PKD is activated differently via CERT at the TGN: in the short distance shuttle model, the flow from state 1 to state 2 is tuned by both the amount of ceramide-carrying unphosphorylated CERT at the ER (state 5) and the amount of PI4KIIIβp (state 4), because PI4KIIIβp recruits CERT from the ER to the TGN by producing PI4P; in the neck-swinging model, the flow from state 1 to state 2 is only tuned by CERT in the double-bound state 7. The graphs represent all the interactions in systems (2.1) and (2.2), apart from external inflows *s*_*i*_ and species self-degradation.

In this paper, the flow-inducing backbone of the models (2.1) and (2.2), captured by the graphs in figure 5, is exploited to get parameter-free insight into the structure of the interactions, so as to understand the design principles underlying the two models and compare their structures.

### 2.3. Algorithmic methods: structural steady-state input–output influences

This section surveys structural steady-state influences in dynamical systems; additional details are in the electronic supplementary material, §2. The algorithm proposed in Giordano *et al.* [53] to efficiently compute structural influences has been used to obtain the structural results in §§3.3 and 3.4.

Consider a generic nonlinear system

2.4 $$\dot{x}(t)=f(x(t),u(t))$$

and

2.5 y(t)=g(x(t)),

with an *output* *y*, an *input* *u* and an asymptotically stable equilibrium $\left(\bar{x},\bar{u}\right)$, such that $f(\bar{x},\bar{u})=0$. Then, the *steady-state input–output influence* [53,70] is the ensuing variation of the steady-state value of the system output, upon a persistent perturbation due to a constant input applied to the system. Its sign can be computed based on the linear approximation of the nonlinear system in a neighbourhood of the equilibrium $\bar{x}$, with $z(t)=x(t)-\bar{x}$, $v(t)=u(t)-\bar{u}$, $w(t)=y(t)-\bar{y}$:

$$\dot{z}(t)=Jz(t)+Ev(t)$$

and

w(t)=Hz(t),

where *J* is the Jacobian matrix computed at the equilibrium, while *E* and *H* are a column and a row vector that represent, respectively, how the input acts on the system state and how the output depends on the system state in the linearized system. Then, the sign of the steady-state input–output influence [53] turns out to be

$$\text{sign}\;\left(\det\left[\begin{array}{cc} -J & -E\\ H & 0 \end{array}\right]\right).$$

A steady-state input–output influence is *structurally signed* if, upon a perturbation due to a constant input *u*, the ensuing variation of the steady-state output value has always the same sign as the input (‘+’, structurally positive influence), always the opposite sign (‘−’, structurally negative influence) or is always zero (‘0’, the so-called perfect adaptation [71–73], which is fundamental in many biological systems [74–77]), *for any feasible choice of the parameter values*; it is undetermined if the behaviour is *parameter-dependent* (‘?’).

When a persistent additive input is applied to a single state equation and a single state variable is taken as the system output, the results for all the possible input–output pair combinations can be visualized in the *structural influence matrix* *Σ*, whose (*i*, *j*)th entry *Σ*_*ij*_ expresses the sign of the overall steady-state influence on the *i*th system variable of an external persistent additive input applied to the dynamic equation of the *j*th system variable. The entries of matrix *Σ* can be computed as $\Sigma_{ij}=\text{sign}(\det[\begin{array}{cc} -J & -E_{i}\\ H_{i} & 0 \end{array}])$, where *E* = *E*_*j*_ is a column vector whose entries are all zero but the *j*th, which is 1, and *H* = *H*_*i*_ is a row vector whose entries are all zero but the *i*th, which is 1.

To structurally evaluate the sign of steady-state input–output influences, the vertex algorithm proposed in Giordano *et al.* [53] can be applied if the system admits the so-called BDC-decomposition [44,45,49,53], namely, its Jacobian can be written in the form $J=\sum_{i=1}^{q}d_{i}M_{i}$, where the matrices *M*_*i*_ have rank one and the scalars *d*_*i*_ are positive and related to the system partial derivatives (see [44,53] for details). The outcome of the algorithm is as follows:

— ‘+’ if the influence is structurally positive, for any feasible choice of the parameters;
— ‘0’ if there is structurally perfect adaptation for any feasible choice of the parameters;
— ‘−’ if the influence is structurally negative, for any feasible choice of the parameters;
— ‘?’ if the influence can have a different sign depending on the chosen parameters.

### 2.4. Numerical methods

The computation of the structural steady-state influence with the algorithm discussed in §2.3 has been implemented in Matlab.

Matlab has been used also to numerically simulate the behaviour of systems (2.1) and (2.2), namely, compute the value of the system variables over time, starting from given initial conditions, until a steady state is reached. In particular, the solutions of the systems of ODEs have been computed using the Matlab function ode15s, well suited for stiff differential equations like those in (2.1) and (2.2). To simulate the systems, functions and parameters have been chosen as in Weber *et al.* [27].

## 3. Results

### 3.1. Comments on the models' flow-inducing structure

The two systems (2.1) and (2.2) can be seen as *flow-inducing networks*, a concept recently introduced in Giordano & Blanchini [54] (see §2.2). The interaction networks for the two models, seen as flow-inducing systems, are visualized in figure 5.

A flow-inducing network is the interconnection of various subsystems, or modules: indeed, both systems (2.1) and (2.2) can be seen as the interplay of three modules, involving variables *x*_1_-*x*_2_ (PKD-subsystem), *x*_3_-*x*_4_ (PI4KIIIβ-subsystem) and *x*_5_-*x*_6_-*x*_7_ (CERT-subsystem). Each module is enclosed in a dashed green box in figure 5. In a flow-inducing network, mass flows involve exclusively variables that belong to the same subsystem (cf. the blue arrow head arcs in figure 5); some mass flows among the variables within a subsystem are spontaneous (those from *x*_2_ to *x*_1_ and from *x*_4_ to *x*_3_ in both systems (2.1) and (2.2); that from *x*_6_ to *x*_5_ in the short distance shuttle system (2.1); those from *x*_5_ to *x*_6_ and from *x*_6_ to *x*_5_ in the neck-swinging system (2.2)); other mass flows among the variables within a subsystem are tuned by the value of variables that belong to other subsystems, which creates coupling effects among the subsystems (represented by the red hammer head links in figure 5).

In particular, in the short distance shuttle system (2.1): *x*_2_, in the first subsystem, induces the flow from *x*_3_ to *x*_4_ in the second subsystem; *x*_2_, in the first subsystem, induces the flow from *x*_7_ to *x*_6_ in the third subsystem; *x*_4_, in the second subsystem, induces the flow from *x*_1_ to *x*_2_ in the first subsystem; *x*_4_, in the second subsystem, induces the flow from *x*_5_ to *x*_7_ in the third subsystem; and *x*_5_, in the third subsystem, induces the flow from *x*_1_ to *x*_2_ in the first subsystem. Conversely, in the short distance shuttle system (2.2): *x*_2_, in the first subsystem, induces the flow from *x*_3_ to *x*_4_ in the second subsystem; *x*_2_, in the first subsystem, induces the flow from *x*_7_ to *x*_6_ in the third subsystem; *x*_4_, in the second subsystem, induces the flow from *x*_6_ to *x*_7_ in the third subsystem; and *x*_7_, in the third subsystem, induces the flow from *x*_1_ to *x*_2_ in the first subsystem. These flow-inducing effects are a fundamental aspect to capture how CERT-mediated ceramide transfer can be tuned and governed by the key molecules PKD and PI4KIIIβ.

In the PKD-subsystem, the only difference lies in the feedback term from the other subsystems, which in (2.1) depends on *x*_4_ and *x*_5_, in (2.2) on *x*_7_ only, while the PI4KIIIβ-subsystem is described by the same equations in both models. The important difference is in the form of the CERT-subsystem and in the PKD–CERT feedback relation. In both cases, (i) CERT-mediated transport has a positive effect on the activity of PKD; and (ii) active PKD has two main roles: it detaches CERT from the TGN membrane and it activates PI4KIIIβ. Yet, the outcome is completely different.

In (2.1), the CERT-subsystem has a circular structure: extraction and delivery of ceramide *requires* the temporary detachment of CERT from the TGN. Hence, the larger the circular flow involving CERTaER, CERTp and CERTaTGN, the more ceramide will be transferred. Active PKD favours this ‘circulation’ *directly*, by steering the transition of CERT from state 7 to state 6, and *indirectly*, by promoting the activation of PI4KIIIβ, which in turn steers the transition from state 5 to state 7. Therefore, active PKD sustains ceramide transfer in a coherent feed-forward loop motif [32,78,79], and the overall PKD-CERT feedback loop, as suggested in Weber *et al.* [27], is *positive*.

In (2.2), the CERT-subsystem has a cascaded double-switch structure: the process can be switched off by active PKD and switched on by active PI4KIIIβ. As only unphosphorylated CERT that is bound to both membranes can transfer ceramide from the ER to the TGN, the larger the concentration of CERT in this state, the more ceramide will be transferred. Now, the overall PKD–CERT feedback loop does not have a neat sign: active PKD switches off ceramide transfer, but it also activates PI4KIIIβ, which switches on ceramide transfer. Hence, the overall net effect depends on the strength and the time constants of the two antagonistic paths, which form an incoherent feed-forward loop [32,78,79].

### 3.2. Positivity, boundedness, equilibria and Jacobian analysis

The systems (2.1) and (2.2) are positive (the variables, species concentrations, cannot become negative) and bounded (the variables cannot diverge), hence they admit an equilibrium point. In fact, for both systems, the trajectories starting from a non-negative initial condition (*x*(0)≥0, componentwise) lie in the non-negative orthant for all subsequent time instants (*x*(*t*)≥0 ∀*t*); in particular, the trajectories of each subsystem are bounded in a simplex included in the non-negative orthant.

**Proposition 3.1.**

*System* (*2.1*) *and system* (*2.2*) *are both positive systems. For any non-negative initial condition*, *their solutions are bounded in the set*

3.1 $$\begin{array}{c} x_{1}+x_{2}\leq k_{1},\\ x_{3}+x_{4}\leq k_{2},\\ x_{5}+x_{6}+x_{7}\leq k_{3}\\ x_{i}\geq 0,\forall \;i, \end{array}\}$$

*for fixed positive constants k*_1_, *k*_2_ *and* *k*_3_.

**Proof.**

For both systems (2.1) and (2.2), when *x*_*i*_ = 0, with i∈{1,…,7}, the corresponding derivative is ${\dot{x}}_{i}\geq 0$, hence *x*_*i*_ cannot become negative: *x*_*i*_≥0 for all *i*. Therefore, the non-negative orthant is positively invariant, which proves positivity. To prove boundedness, the sum of the derivatives for each subsystem can be considered (which, interestingly, is the same for both models). For the PKD-subsystem,

$${\dot{x}}_{1}+{\dot{x}}_{2}=s_{1}-\alpha x_{1}-\beta x_{2}<s_{1}-\min\{\alpha ,\beta \}(x_{1}+x_{2}).$$

As *s*_1_ is constant, ${\dot{x}}_{1}+{\dot{x}}_{2}$ becomes negative when *x*_1_ + *x*_2_ exceeds a threshold value *k*_1_, hence *x*_1_ + *x*_2_ ≤ *k*_1_. Analogously, for the PI4KIIIβ-subsystem,

$${\dot{x}}_{3}+{\dot{x}}_{4}=s_{3}-\gamma x_{3}-\delta x_{4}<s_{3}-\min\{\gamma ,\delta \}(x_{3}+x_{4}),$$

thus *x*_3_ + *x*_4_ ≤ *k*_2_, while for the CERT-subsystem

$${\dot{x}}_{5}+{\dot{x}}_{6}+{\dot{x}}_{7}=s_{5}-\varepsilon x_{5}-\zeta x_{6}-\eta x_{7}<s_{5}-\min\{\varepsilon ,\zeta ,\eta \}(x_{5}+x_{6}+x_{7}),$$

therefore *x*_5_ + *x*_6_ + *x*_7_ ≤ *k*_3_. Hence, the state trajectories of each subsystem are bounded in a simplex in the non-negative orthant. ▪

As the solutions are globally asymptotically bounded in a (convex and compact) set S, an equilibrium point must exist inside S [80–82]. This proves the existence of an equilibrium. Numerical simulations show that, with reasonable choices of the functions and of the parameters (cf. [27]), the equilibrium point is unique and stable, as expected.

The Jacobian matrices associated with the linearization of the two systems around the equilibrium are here reported: the Jacobian *J*_SDS_ for system (2.1) is in table 1, top, while the Jacobian *J*_NS_ for system (2.2) is in table 1, bottom. In both cases, in view of assumption 2.1, all of the Greek letters denote positive values.

**Table 1.** Jacobian matrix of the short distance shuttle model, *J*_SDS_ (top), and of the neck-swinging model, *J*_NS_ (bottom). Monotone subsystems are squared in green (and correspond to the modules inside the dashed green boxes in figure 5), their interactions in red (corresponding to the hammer head red arrows in figure 5). For *J*_SDS_, $\vartheta =h({\bar{x}}_{4},{\bar{x}}_{5})$, $\mu ={\bar{x}}_{1}.\partial h(x_{4},x_{5})/\partial x_{5}{|}_{\left({\bar{x}}_{4},{\bar{x}}_{5}\right)}$, $\nu ={\bar{x}}_{1}.\partial h(x_{4},x_{5})/\partial x_{4}{|}_{\left({\bar{x}}_{4},{\bar{x}}_{5}\right)}$, $\xi =f_{1}({\bar{u}}_{1})$, $\pi =f_{2}({\bar{u}}_{2})$, $\rho ={\bar{x}}_{3}.\partial g_{1}(x_{2})/\partial x_{2}{|}_{{\bar{x}}_{2}}$, $\sigma =g_{1}({\bar{x}}_{2})$, $\tau ={\bar{x}}_{7}.\partial g_{2}(x_{2})/\partial x_{2}{|}_{{\bar{x}}_{2}}$, $\omega =g_{2}({\bar{x}}_{2})$, $\psi ={\bar{x}}_{5}.\partial g_{4}(x_{4})/\partial x_{4}{|}_{{\bar{x}}_{4}}$, $\varphi =g_{4}({\bar{x}}_{4})$. For *J*_NS_, $\vartheta =g_{7}({\bar{x}}_{7})$, $\mu ={\bar{x}}_{1}.\partial g_{7}(x_{7})/\partial x_{7}{|}_{{\bar{x}}_{7}}$, $\xi =f_{1}({\bar{u}}_{1})$, $\pi =f_{2}({\bar{u}}_{2})$, $\rho ={\bar{x}}_{3}.\partial g_{1}(x_{2})/\partial x_{2}{|}_{{\bar{x}}_{2}}$, $\sigma =g_{1}({\bar{x}}_{2})$, $\tau ={\bar{x}}_{7}.\partial g_{2}(x_{2})/\partial x_{2}{|}_{{\bar{x}}_{2}}$, $\omega =g_{2}({\bar{x}}_{2})$, $\psi ={\bar{x}}_{6}.\partial g_{4}(x_{4})/\partial x_{4}{|}_{{\bar{x}}_{4}}$, $\varphi =g_{4}({\bar{x}}_{4})$. All Greek letters denote positive values.

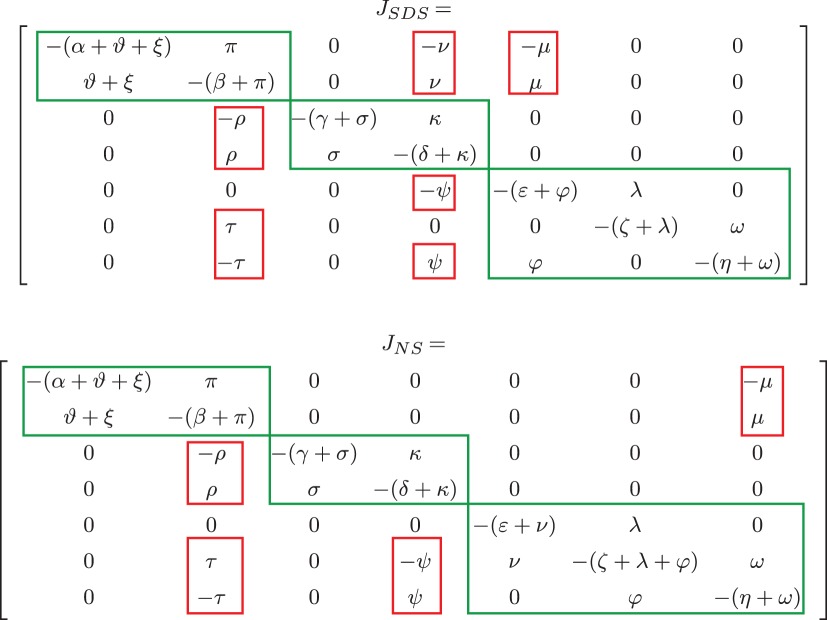

Both the models are the interconnection of monotone subsystems [83–86]: the diagonal blocks in the Jacobian matrix, squared in green in table 1, are Metzler matrices (matrix *M* is Metzler if it has non-negative off-diagonal entries: [*M*]_*ij*_≥0 for all *i*≠*j*). Each subsystem is also structurally stable, because the corresponding Metzler matrix is column diagonally dominant ($|M_{ii}|\geq \sum_{j}|M_{ji}|$) and has negative diagonal entries (*M*_*ii*_ < 0,  ∀*i*). The flow-inducing interactions that constitute the coupling among the subsystems, leading to mass transfer within a subsystem that is tuned by a variable in another subsystem, are squared in red in table 1.

### 3.3. Ceramide-transfer protein-mediated transfer relies on structurally signed input–output influences

Systems (2.1) and (2.2) admit a BDC-decomposition (cf. §2.3 and §2 of the electronic supplementary material): in fact, the Jacobians in table 1 can be written in the form $\sum_{i=1}^{q}d_{i}M_{i}$, where *d*_*i*_ correspond to the Greek letters and *M*_*i*_ are rank-one matrices. Therefore, the vertex algorithm proposed in Giordano *et al.* [53] can be applied to structurally evaluate the sign of steady-state input–output influences in these systems and to compute the influence matrices corresponding to the two models, which are

3.2 $$\Sigma_{\mathrm{SDS}}=\left[\begin{array}{ccccccc} ? & ? & ? & ? & - & - & -\\ + & + & ? & ? & + & + & +\\ - & - & ? & ? & - & - & -\\ + & + & ? & ? & + & + & +\\ ? & ? & ? & ? & ? & ? & ?\\ + & + & ? & ? & ? & ? & ?\\ ? & ? & ? & ? & ? & ? & ? \end{array}\right]\;\mathrm{and}\;\Sigma_{\mathrm{NS}}=\left[\begin{array}{ccccccc} ? & ? & - & - & - & - & -\\ + & + & + & + & + & + & +\\ - & - & ? & ? & - & - & -\\ + & + & + & + & + & + & +\\ ? & ? & - & - & ? & ? & ?\\ ? & ? & - & - & ? & ? & ?\\ ? & ? & + & + & + & + & + \end{array}\right].$$

When studying two different models aimed at describing the same phenomenon, it is interesting to compare the outcomes with each other, and also with possible experimental results. Consistency can be investigated as follows: two signed entries are strongly consistent if they have the same sign (namely, both ‘+’, both ‘−’, or both ‘0’); weakly consistent if there is no contradiction because at least one of the two signs is undetermined, ‘?’; inconsistent if there is contradiction (for instance, one sign is ‘+’ and the other is ‘−’, or ‘0’). The following is a formal definition.

**Definition 3.2.**

Consider two different models of the same phenomenon, model *A* and model *B* (or, equivalently, model *A* and the experimental observations *B*), both with input *i* and output *o*. The two *input–output structural influences* *σ*^*A*^_*io*_ and *σ*^*B*^_*io*_, computed according to the two different models, are *strongly consistent* if (*σ*^*A*^_*io*_, *σ*^*B*^_*io*_)∈ {( + , + );( − , − );(0, 0)}, *weakly consistent* if (*σ*^*A*^_*io*_, *σ*^*B*^_*io*_)∈{( + , ?);(?, + );( − , ?);(?, − );(0, ?);(?, 0);(?, ?)}, *inconsistent* otherwise. The two *structural influence matrices* *Σ*^*A*^ and *Σ*^*B*^ corresponding to model *A* and model *B* (assumed to have the same variables representing the same entities) are *strongly consistent* if all possible pairs of corresponding entries (*Σ*^*A*^_*ij*_, *Σ*^*B*^_*ij*_) are strongly consistent, *weakly consistent* if all possible pairs of corresponding entries (*Σ*^*A*^_*ij*_, *Σ*^*B*^_*ij*_) are either strongly or weakly consistent, and *inconsistent* otherwise.

The difference between strong and weak consistency is subtle but fundamental. Weak consistency is not inconsistency, but can lead to inconsistency if additional specifications are introduced. While strong consistency is guaranteed for any possible choice of the parameters, hence also for any restriction to a subset of the parameters, weak inconsistency may no longer hold if further information on the models is available and thus the parameter space is restricted. In fact, weak consistency involves either a sign-determined value compared to non-determined value, or two non-determined values. Yet, when restricting to a subset of the parameter space, non-determined values may become determined, and the two models may become inconsistent. Consider, for instance, weak consistency between ‘?’, for one model, and ‘+’, for the other. If additional information on the first model leads to a restriction of the parameter space where the ‘?’ becomes ‘−’, this leads to inconsistency.

The two structural influence matrices *Σ*_SDS_ and *Σ*_NS_ are *weakly consistent*: there is no pair of corresponding entries having inconsistent signs. In particular, 18 out of 49 entries are *strongly consistent* and represent signed behaviours that are crucial for the system to perform its function: a persistent input that feeds active (respectively, inactive) PKD will increase (respectively, decrease) the concentration of CERT in all its three states, thus revealing that the presence of active PKD is fundamental for ceramide transfer to occur, and will also increase the concentration of PKD, both active and inactive; a persistent input that feeds active (respectively, inactive) PI4KIIIβ will increase (respectively, decrease) the concentration of CERT in all its three states, thus revealing that also the presence of active PI4KIIIβ is fundamental for ceramide transfer, and will also increase (respectively, decrease) the concentration of PI4KIIIβ, both active and inactive.

Also the structural effect on the steady-state value of each variable due to a persistent step input applied through *u*_1_ and *u*_2_ in systems (2.1) and (2.2) can be computed with the vertex algorithm in Giordano *et al.* [53]:

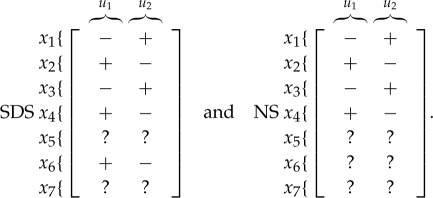
Expectedly, the signed influence of *u*_2_ is always opposite to that of *u*_1_. Again, the results are *weakly consistent*, and the first four of the seven entries are *strongly consistent*: in fact, in both models it is fundamental that a persistent input *u*_1_ always increases the amount of active PKD and active PI4KIIIβ, decreasing the amount of their inactive counterparts.

### 3.4. Ceramide-transfer protein-mediated transfer is a structurally tunable flow-inducing mechanism

The analysis tools provided in Giordano *et al.* [53] for structural input–output influences can provide more insight into the *structural* flow-induction mechanism that regulates ceramide transfer. First, it is necessary to define an output that is proportional to the amount of ceramide actually transferred.

For the short distance shuttle model, as *the whole cyclic reaction scheme is required to sustain ceramide transfer*, the natural output is the flow variable

3.3 $$\Phi_{\mathrm{SDS}}=\lambda x_{6}+x_{7}g_{2}(x_{2})+x_{5}g_{4}(x_{4}),$$

which suitably quantifies the circular flow involving CERTaER, CERTp and CERTaTGN (namely, the sum of the flows involved in the smallest loop that includes TGN-bound CERT). The structural influence on *Φ*_SDS_ of step-like persistent variations in the input *u*_*i*_, *i* = 1, 2, and of a step-like persistent input affecting the equation of variable *x*_*i*_, *i* = 1, …, 7, computed based on the algorithm in Giordano *et al.* [53], is summarized as follows (details on the computation are in the electronic supplementary material, §4):

$$\begin{array}{c} \Phi_{\mathrm{SDS}}\{ \end{array}\;[\;\overset{u_{1}}{\overbrace{\begin{array}{c} + \end{array}}}\;\overset{u_{2}}{\overbrace{\begin{array}{c} - \end{array}}}\;\overset{x_{1}}{\overbrace{\begin{array}{c} + \end{array}}}\;\overset{x_{2}}{\overbrace{\begin{array}{c} + \end{array}}}\;\overset{x_{3}}{\overbrace{\begin{array}{c} ? \end{array}}}\;\overset{x_{4}}{\overbrace{\begin{array}{c} ? \end{array}}}\;\overset{x_{5}}{\overbrace{\begin{array}{c} ? \end{array}}}\;\overset{x_{6}}{\overbrace{\begin{array}{c} ? \end{array}}}\;\overset{x_{7}}{\overbrace{\begin{array}{c} ? \end{array}}}\;].$$

The steady-state influence from any input increasing the concentration *x*_2_ of active PKD (either due to a persistent transfer *u*_1_ or due to a persistent injection directly applied to the equation of *x*_2_) to the output *Φ*_SDS_ is structurally positive: the steady-state flow always increases. Applying a persistent input to the equation of *x*_1_ (inactive PKD) yields the same structurally positive influence. Of course, the input *u*_2_ has an opposite effect. Then, the system is *structurally designed so that the abundance of active PKD regulates the CERT-mediated flow of ceramide*. This is consistent with the observation in Weber *et al.* [27] that PKD is the dominant regulator of CERT-dependent ceramide trafficking.

For the neck-swinging model, conversely, *x*_7_ (the concentration of CERT molecules in the double-bound state) is an appropriate output to quantify ceramide transfer, because CERT-mediated ceramide trafficking can occur only when CERT is bound to both the ER and the TGN. A different output could be the flow variable

3.4 $$\Phi_{\mathrm{NS}}=x_{6}g_{4}(x_{4})+x_{7}g_{2}(x_{2}),$$

which quantifies the circular flow involving CERTaERTGN and CERTp (namely, analogously to the short distance shuttle case, the sum of the flows involved in the smallest loop that includes TGN-bound CERT). The steady-state input–output influences computed with the algorithm in Giordano *et al.* [53] can be summarized as follows (details on the computation are in the electronic supplementary material, §4):

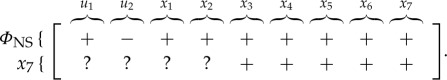
If *x*_7_ is taken as an output, the influence due to a persistent input applied to the equation of variables *x*_3_, *x*_4_, *x*_5_, *x*_6_ and *x*_7_ is always structurally positive. However, inputs applied to the equation of variables *x*_1_ and *x*_2_, as well as inputs *u*_1_ and *u*_2_, yield an undetermined structural influence. So, PI4KIIIβ has a structurally positive effect on ceramide transfer. Conversely, PKD has no structural effect on ceramide transfer in this model.

Conversely, if *Φ*_NS_ is taken as an output, the influence from PKD is structurally positive: increasing the concentration of active PKD due to a persistent input leads to an increase in the steady-state flow, regardless of the value of the parameters and regardless of the choice of the functions in the model (provided that they satisfy assumption 2.1). Interestingly, in this case the same behaviour occurs if a persistent input is applied not only to the equation of *x*_1_, but also to the equation of *x*_2_, *x*_3_, *x*_4_, *x*_5_, *x*_6_ and *x*_7_ (and, therefore, to any group of more variables). The system seems therefore *structurally designed to increase mobility of CERT in the cytosol*, which is indeed represented by the variable *Φ*_NS_.

It seems therefore that *also in the neck-swinging model* CERT is expected not to remain attached to both membranes in the same location for a long time, but to frequently unbind, travel in the cytosol and then bind again to both the ER and the TGN. Why is this increased mobility needed? The following hypothesis can be suggested: if CERT remains bound to both the ER and the TGN in the same location, the area will be soon depleted of ceramide. Conversely, increasing CERT mobility in the cytosol could help it detach and bind again where there is much more ceramide to be transferred: this would make the transfer mechanism more efficient. This aspect is significant because it suggests that PKD can actually increase ceramide transfer even in the neck-swinging model, because it induces detachment of CERT from the TGN and is thus responsible for its increased mobility.

**Remark 3.3.**

The outputs in equations (3.3) and (3.4) are a natural choice to represent CERT flow, after embedding of the two models in the flow-inducing framework. Indeed, *Φ*_SDS_ and *Φ*_NS_ represent the circular flows that involve TGN-bound CERT (state 7), as shown in the graphs in figures 3 and 5: they are the sum of the flow components associated with the arcs forming the minimal-length loop that involves the variable *x*_7_ in the two graphs. The results above point out that, in the short distance shuttle model, ceramide transport is due to *circulation* (not to the fraction of CERT in the various states, but to its flow between one state and the other), while in the neck-swinging model what mainly counts is the *fraction* of CERT in state 7, which is the transport-active state.

The structural results point out an interesting difference between the two models. In the short distance shuttle model, PKD is the only *structural* regulator of CERT-mediated ceramide transfer, and has a structurally positive effect on ceramide flow. Conversely, in the neck-swinging model, a structurally positive regulation is due to PI4KIIIβ; PKD cannot provide a structurally signed regulation, due to the presence of an incoherent feed-forward loop [32] where it directly inhibits ceramide transfer and indirectly promotes it, through PI4KIIIβ. Yet, even in the neck-swinging model, PKD has a structurally positive effect on the mobility of CERT in the cytosol.

### 3.5. Numerical simulations

This section reports simulations of the behaviour of systems (2.1) and (2.2), for functions *f*_*i*_, *g*_*i*_ and *h* and parameters chosen as in Weber *et al.* [27] (cf. §2.1). For the sake of completeness, we report here the systems achieved with the choice of functions and parameters as in Weber *et al.* [27]: the short distance shuttle model becomes

$$\begin{array}{rl} {\dot{x}}_{1} & =-p_{11}x_{1}\frac{p_{31}x_{5}x_{4}/(x_{4}+m_{31})}{\left(m_{11}+p_{31}x_{5}x_{4})/(x_{4}+m_{31}\right)}-p_{12}(1+u)x_{1}+p_{13}x_{2}-a_{11}x_{1}+s_{1},\\ {\dot{x}}_{2} & =p_{11}x_{1}\frac{p_{31}x_{5}x_{4}/(x_{4}+m_{31})}{\left(m_{11}+p_{31}x_{5}x_{4})/(x_{4}+m_{31}\right)}+p_{12}(1+u)x_{1}-p_{13}x_{2}-a_{12}x_{2},\\ {\dot{x}}_{3} & =p_{21}x_{4}-p_{22}\frac{x_{3}x_{2}}{x_{2}+m_{22}}-a_{21}x_{3}+s_{3},\\ {\dot{x}}_{4} & =-p_{21}x_{4}+p_{22}\frac{x_{3}x_{2}}{x_{2}+m_{22}}-a_{22}x_{4},\\ {\dot{x}}_{5} & =-p_{31}\frac{x_{5}x_{4}}{x_{4}+m_{31}}+p_{32}x_{6}-a_{31}x_{5}+s_{5},\\ {\dot{x}}_{6} & =-p_{32}x_{6}+p_{33}\frac{x_{7}x_{2}}{x_{2}+m_{33}}-a_{32}x_{6}\\ \;\mathrm{and}\;\;{\dot{x}}_{7} & =p_{31}\frac{x_{5}x_{4}}{x_{4}+m_{31}}-p_{33}\frac{x_{7}x_{2}}{x_{2}+m_{33}}-a_{33}x_{7}, \end{array}$$

where the parameter values are *p*_11_ = 0.6467; *p*_12_ = 0.0507; *p*_13_ = 0.2064; *m*_11_ = 2.3412 × 10^7^; *s*_1_ = 4.8511 × 10^5^; *a*_11_ = 0.9933; *a*_12_ = 9.2877; *p*_21_ = 8.1888; *p*_22_ = 1.8621; *m*_22_ = 7191.9; *s*_3_ = 2.0894 × 10^6^; *a*_21_ = 1.3142; *a*_22_ = 0.0030; *p*_31_ = 636.5506; *p*_32_ = 4.7598; *p*_33_ = 12861; *m*_31_ = 4.5024 × 10^9^; *m*_33_ = 7.6925 × 10^6^; *s*_5_ = 139.0702; *a*_31_ = 2.2868 × 10^−4^; *a*_32_ = 7.8218 × 10^−4^; *a*_33_ = 3.2251 × 10^−4^, while the neck-swinging model becomes

$$\begin{array}{rl} {\dot{x}}_{1} & =-p_{11}x_{1}\frac{x_{7}}{x_{7}+m_{11}}-p_{12}(1+u)x_{1}+p_{13}x_{2}-a_{11}x_{1}+s_{1},\\ {\dot{x}}_{2} & =p_{11}x_{1}\frac{x_{7}}{x_{7}+m_{11}}+p_{12}(1+u)x_{1}-p_{13}x_{2}-a_{12}x_{2},\\ {\dot{x}}_{3} & =p_{21}x_{4}-p_{22}x_{3}\frac{x_{2}}{x_{2}+m_{22}}-a_{21}x_{3}+s_{3},\\ {\dot{x}}_{4} & =-p_{21}x_{4}+p_{22}x_{3}\frac{x_{2}}{x_{2}+m_{22}}-a_{22}x_{4},\\ {\dot{x}}_{5} & =-p_{31}x_{5}+p_{32}x_{6}-a_{31}x_{5}+s_{5},\\ {\dot{x}}_{6} & =p_{31}x_{5}-p_{32}x_{6}-p_{33}x_{6}\frac{x_{4}}{m_{31}+x_{4}}+p_{34}x_{7}\frac{x_{2}}{x_{2}+m_{33}}-a_{32}x_{6}\\ \;\mathrm{and}\;\;{\dot{x}}_{7} & =p_{33}x_{6}\frac{x_{4}}{m_{31}+x_{4}}-p_{34}x_{7}\frac{x_{2}}{x_{2}+m_{33}}-a_{33}x_{7}, \end{array}$$

where the parameter values are *p*_11_ = 1.3493; *p*_12_ = 7.1440 × 10^−4^; *p*_13_ = 3.1170; *m*_11_ = 5.9974 × 10^5^; *s*_1_ = 6.2447 × 10^5^; *a*_13_ = 1.2879; *a*_14_ = 4.2273; *a*_11_ = 0.9933; *a*_12_ = 9.2877; *p*_21_ = 0.5782; *p*_22_ = 1.1462; *m*_22_ = 2.1550 × 10^4^; *s*_3_ = 6.9681 × 10^5^; *a*_21_ = 0.0903; *a*_22_ = 8.7353; *p*_31_ = 3.1330 × 10^−4^; *p*_32_ = 0.0012; *p*_33_ = 207.5791; *p*_34_ = 516.5629; *m*_31_ = 6.5757 × 10^6^; *m*_33_ = 7.8586 × 10^6^; *s*_5_ = 2122.7; *a*_31_ = 0.0046; *a*_32_ = 0.0043; *a*_33_ = 7.1526 × 10^−4^.

The evolution of the two systems above, with given initial conditions (reported in the figure captions), is computed until a steady state is reached; then, a persistent positive step input is applied and the system evolves to a new steady state. The sign of the difference between the old and the new steady states is the signed input–output influence.

The sign of the variation in the steady state of *x*_*i*_ due to an additive step input applied to the equation of variable *x*_*j*_ is given by the (*i*, *j*)th entry of the following matrices:
3.5
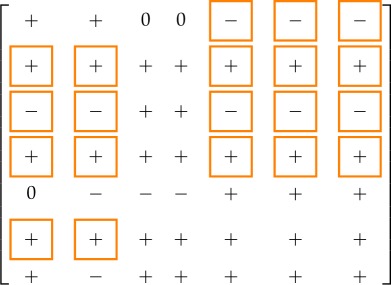
for the short distance shuttle system and
3.6
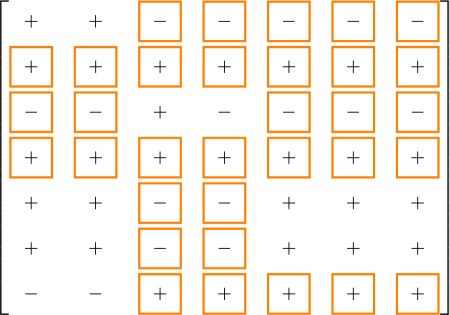
for the neck-swinging system. The entries squared in orange are those for which the influence is *structurally signed* according to the *structural influence matrix*: for all of these entries, the simulation results are consistent with the expected sign.

**Figure 6.**
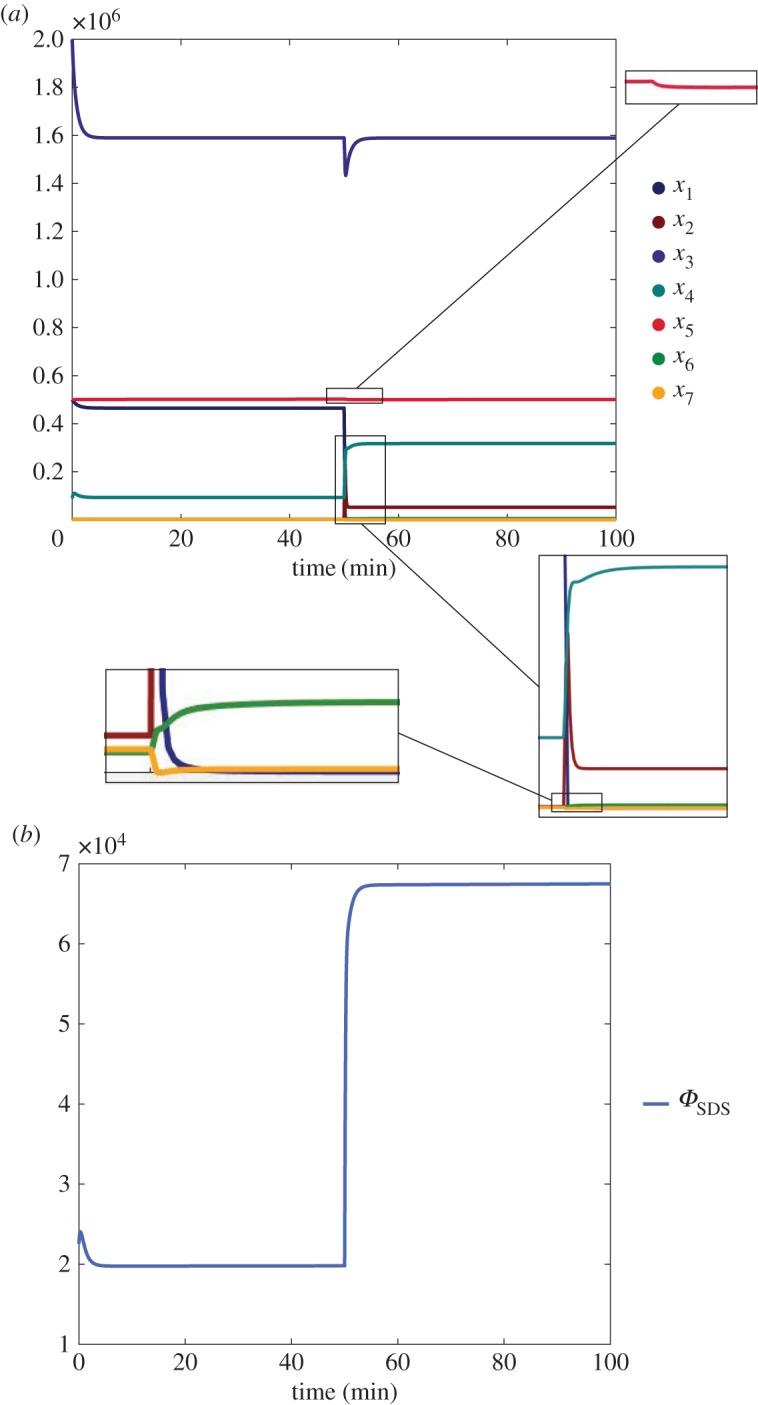
Time evolution of the *short distance shuttle* system (2.1), when *u*_1_ is suddenly increased from 0 to 10^5^ at time t=50 min. Initial conditions: *x*_1_(0) = 5 × 10^5^, *x*_2_(0) = 2 × 10^3^, *x*_3_(0) = 2 × 10^6^, *x*_4_(0) = 9 × 10^4^, *x*_5_(0) = 5 × 10^5^, *x*_6_(0) = 2 × 10^3^, *x*_7_(0) = 2 × 10^3^ (molecules/cell). (*a*) State variables. (*b*) Flow variable.

**Figure 7.**
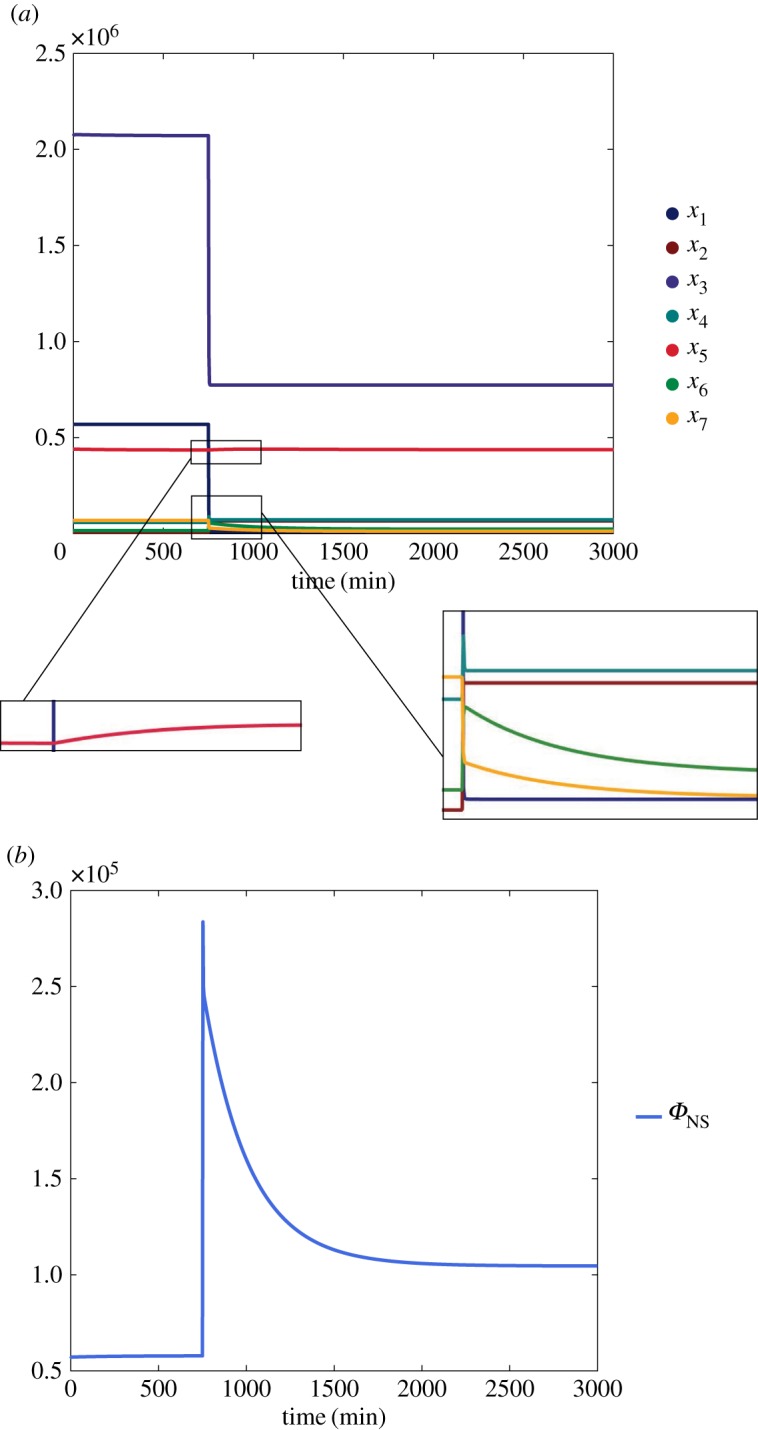
Time evolution of the *neck-swinging* system (2.2), when *u*_1_ is suddenly increased from 0 to 10^5^ at time *t* = 750 min. Initial conditions: *x*_1_(0) = 5.7 × 10^5^, *x*_2_(0) = 6.4 × 10^3^, *x*_3_(0) = 2.07 × 10^6^, *x*_4_(0) = 5.8 × 10^4^, *x*_5_(0) = 4.4 × 10^5^, *x*_6_(0) = 1.6 × 10^3^, *x*_7_(0) = 6.8 × 10^4^ (molecules/cell). (*a*) State variables. (*b*) Flow variable.

The following tables summarize the effect of a persistent step input applied through *u*_1_ and *u*_2_: for the short distance shuttle system,

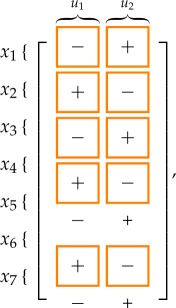
while for the neck-swinging system

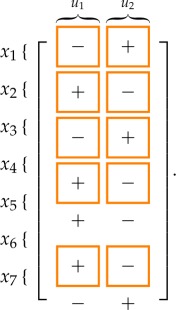
Again, all the structurally sign-determined entries, squared in orange, exactly match the expectation. The time evolution of the state variables is shown for the short distance shuttle system in figure 6*a*, where a persistent step input has been applied through *u*_1_ at the time t=50 min, and for the neck-swinging system in figure 7*a*, where a persistent step input has been applied through *u*_1_ at the time t=750 min.

Also the influence on the flow variable of step inputs, either applied through *u*_1_ and *u*_2_ or added to the equation of the variables *x*_*j*_, has been computed and is reported for both the short distance shuttle system

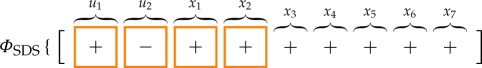
and the neck-swinging system

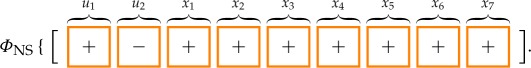
The input–output influences that are structurally signed according to the theoretical results, squared in orange, are consistent with the expectation. As an example, the time evolution of the flow variable when a step input is applied through *u*_1_ is shown in figure 6*b* for the short distance shuttle system, and in figure 7*b* for the neck-swinging system.

## 4. Concluding discussion

This work has analysed with a *structural approach* two dynamical systems that describe PKD–PI4KIIIβ–CERT interactions at the *trans*-Golgi network in mammalian cells, first proposed in Weber *et al.* [27], which mathematically represent the mechanism of CERT-mediated ceramide transfer as a ‘short distance shuttle’ [12,24] or as a ‘neck-swinging’ process [3,14,24]. The structural analysis has revealed features that inherently distinguish the two models, regardless of the chosen parameter values, and motif-based [32,39] mechanisms that explain the structurally different functioning of the two models.

In particular, in the *short distance shuttle* model, active PKD is the only structural regulator of CERT-mediated ceramide transfer and its action is enforced through a structural coherent feed-forward loop [32,78,79], which allows PKD to steer ceramide transfer both indirectly, through PI4KIIIβ, and directly. The flow variable that quantifies ceramide transfer is structurally tuned by inputs that affect the concentration of active PKD.

In the *neck-swinging* model, on the other hand, PI4KIIIβ is the only structural regulator of CERT-mediated ceramide transfer. The effect of PKD on CERT activity is not clear, because PKD is involved in an incoherent feed-forward loop [32,78,79]: it steers ceramide transfer indirectly, by activating PI4KIIIβ, but reduces it directly. The effect of this incoherent feed-forward loop can be seen in the time evolution of the flow variable that quantifies CERT mobility in the cytosol, simulated in figure 7*b*, which presents a pulse-like trace with a large spike: spiking behaviours are typical in the presence of incoherent feed-forward motifs [32]. Even though CERT-mediated transfer of ceramide is possible exclusively when CERT is bound to both the ER and the TGN, the system seems structurally designed to have a high mobility of CERT in the cytosol, because a persistent input applied to any of the variables induces a structural increase of the associated flow variable. Why? A possible explanation is that, if CERT remains bound to both the ER and the TGN in the same location, the area will be soon depleted of ceramide, while increasing its mobility in the cytosol could help it detach and bind again where there is much more ceramide to be transferred. This suggests that PKD, by inducing detachment of CERT from the TGN, can increase ceramide transfer even in the neck-swinging model.

It is particularly interesting to reveal clear structural features, which are completely independent of parameters, because they are key aspects of a phenomenon that cannot be modified, at least qualitatively: a structurally positive/negative effect will possibly be weaker or stronger depending on the circumstances, but it will never change sign, no matter how the environmental conditions change. If the sign of this induced variation needs to be preserved at all costs, then it must be crucial for the proper system operation: structural effects are pillars in the system architecture. Conversely, parameter-dependent features reveal aspects where flexibility is fundamental: the system must be able to adapt to the circumstances and turn a positive influence into a negative influence, when the environment changes. In this latter case, sensitivity analysis of the model with respect to parameter values becomes crucial to understand how parameter variations affect the qualitative behaviour.

Those proposed in Weber *et al.* [27] and analysed in this paper are *minimal* models that capture the difference between *two conceptually different mechanisms for CERT-mediated ceramide transfer*, short distance shuttle and neck-swinging, by describing the interactions among PKD, PI4KIIIβ and CERT. A parameter-free *structural approach* that compares the two different mechanisms must rely on simple phenomenological models that capture the essential functioning and allow to draw mathematically grounded conclusions: the models proposed and successfully experimentally validated in Weber *et al.* [27] are perfect for this type of analysis, and well worth investigating in view of their ability to reproduce experimental data very well, even though many interactions are neglected in the explicit model formulation. Their agreement with data shows that these models are able to implicitly consider many additional effects—not explicitly modelled—through their coefficients, when the parameter values are suitably chosen.

The considered model is focused on the representation of the two short distance shuttle and neck-swinging mechanisms, hence simplified with respect to other aspects. Indeed, many other interactions occur at the ER–TGN MCSs. Another fundamental key player is oxysterol binding protein (OSBP) [87], a protein that, like CERT, is attracted to the TGN by the presence of PI4P and is released from the TGN when phosphorylated by PKD; it is in charge of transporting sterols and also transports PI4P from the ER to the TGN [88–90]. PKD phosphorylation of OSBP has been shown to impair CERT localization at the Golgi [88] and OSBP has been reported to promote CERT translocation to the Golgi complex [60,87]. The apparent coupling between CERT and OSBP lipid transfer reactions at ER–Golgi MCSs [87,91,92] is explained [93,94] on the basis of the OSBP cycle: in fact, as it transports PI4P to the TGN, ‘OSBP could set the tempo for the delivery of precursors of complex lipids to the *trans*-Golgi, thereby insuring that the concentrations of cholesterol and sphingolipids increase in parallel along the ER–Golgi interface’ [93, p. 841]. ‘The cellular distribution and the ceramide-transfer activity of the protein CERT might depend on OSBP activity’ [94, p. 946]: by controlling the levels of PI4P at the TGN, OSBP seems to control indirectly the localization and the activity of CERT, which needs PI4P to target the TGN. However, ‘further work is now needed to determine the molecular mechanisms governing OSBP auto-inhibition and their relevance for other FFAT-containing LTPs, including CERT’ [94, p. 945]. Also, recent experimental results [95] interestingly show how the flow of sphingolipids, including ceramide, can control the levels and the dynamic turnover of PI4P at the TGN. Therefore, a very important direction for future work is the construction and experimental validation of new models that explicitly include OSBP and PI4P among their variables, to give a broader picture of the interactions ruling ceramide transfer at the *trans*-Golgi network, beyond the short distance shuttle versus neck-swinging aspect. Given the crucial role of OSBP, this future research direction is particularly relevant: it would be extremely interesting to perform a structural study also of more complex network models also including OSBP.

The ODE models in Weber *et al.* [27] consider the behaviour on an average cellular level and neglect spatial variations (in fact, the model validation in [27] is based on experiments where total quantities are measured, regardless of the molecule locations in the cell). Partial differential equation models could be built to include the effect of spatial inhomogeneities: modelling choices could lead to sensible differences [96,97].

Although the models analysed here have been validated through extensive comparisons with experimental data, and fit very well the experimental traces [27], the conclusions that can be drawn based on the analysis carried out in this paper could actually be used to try and invalidate (one of) the models from a structural standpoint. In fact, the proposed *structural* results need to hold always (regardless of how the model parameters are chosen), *provided that the model is correct*. Therefore, if some experimental results happened to be in contrast with the theoretical predictions that are based on the model structure, this would invalidate the model itself. In particular, structural influences may be used, in combination with experimental data, to *falsify a model*: if the structural sign of an input–output influence is conflicting with experimental observations, then the model is not suitable to describe the observed phenomenon. This can be of great help for experimental investigators when a model must be assessed. For instance, if the theoretical analysis reveals a structurally positive influence of species A on species B in a model, then—for any choice of the system parameters—if a persistent input is added to species A, the new steady-state value of species B must increase with respect to its previous value. If even a single experiment shows that this does not happen, then the model is invalidated. Notably, the whole model is invalidated, regardless of the parameters. Also, in the case of inconsistency between two models, structural results can help design experiments to discriminate between the two models. Assume that model 1 predicts a structurally positive influence of A on B, while model 2 predicts a structurally negative influence. An experiment showing that the influence *can* be positive (respectively, negative) will invalidate model 2 (respectively, 1).

This type of structural insight can be useful not only for a better understanding of biological systems and for model falsification, but also for the construction of artificial biomolecular circuits, in synthetic or bottom-up chemical biology. As an example, the design of the biomolecular oscillators [98,99] has been suggested by mathematically grounded structural considerations.

It is worth stressing that, due to the complexity of the models, the large number of involved species and the huge number of interactions among them, it is virtually impossible to see directly the overall influences among the model variables and to infer structural properties simply by inspecting the system, or the associated graph. The proposed algorithmic approach, instead, provides a systematic tool for structural analysis, which enables model falsification or corroborates model validation, and gives additional insight into the system's inherent design.

In this study, all the theoretical predictions on the structural sign of steady-state influences are *qualitatively* fully consistent both with the numerical simulations of the two models in §3.5 and with the experimental results and extensive simulations reported in Weber *et al.* [27, fig. 4 and fig. S6]. In fact, fig. 4 and fig. S6 in Weber *et al.* [27] show the system reactions to perturbations due to *u*_1_ or *u*_2_, and the *qualitative* changes in the steady states of the variables shown in the graphs therein are fully consistent with the theoretical structural influences.

The insight into the inherent differences between the two models of CERT-mediated transport at the TGN in mammalian cells, achieved with the proposed structural analysis, will hopefully inspire new experiments that could be decisive in understanding which of the two models better describes the actual mechanism. Also, analysing a suite of models achieved by augmenting or perturbing the existing ones could help combine various features of the models to exactly reproduce the qualitative system behaviour, thus generating a new model of the real process that may be different from all the models that have been already proposed; this is another interesting direction for future work.

## Supplementary Material

Supplementary material

## Data accessibility

Additional information is available in the electronic supplementary material.

## References

[1] Dröscher A 1998 Camillo Golgi and the discovery of the Golgi apparatus. Histochem. Cell Biol. 109, 425–430. (doi:10.1007/s004180050245) 9681625 10.1007/s004180050245

[2] Golgi C 1898 Intorno alla struttura delle cellule nervose. Bollettino della Società Medico-Chirurgica di Pavia 13, 3–16.

[3] Hanada K, Kumagai K, Yasuda S, Miura Y, Kawano M, Fukasawa M, Nishijima M 2003 Molecular machinery for non-vesicular trafficking of ceramide. Nature 426, 803–809. (doi:10.1038/nature02188) 14685229 10.1038/nature02188

[4] Mironov AA, Pavelka M 2008 The Golgi apparatus: state of the art 110 years after Camillo Golgi's discovery. Vienna, Austria: Springer.

[5] Mogelsvang S, Marsh BJ, Ladinsky MS, Howell KE 2004 Predicting function from structure: 3D structure studies of the mammalian Golgi complex. Traffic 5, 338–345. (doi:10.1111/j.1398-9219.2004.00186.x) 15086783 10.1111/j.1398-9219.2004.00186.x

[6] Glick BS, Nakano A 2009 Membrane traffic within the Golgi apparatus. Annu. Rev. Cell Dev. Biol. 25, 113–132. (doi:10.1146/annurev.cellbio.24.110707.175421) 19575639 10.1146/annurev.cellbio.24.110707.175421 PMC2877624

[7] Eiseler T, Wille C, Koehler C, Illing A, Seufferlein T 2016 Protein kinase D2 assembles a multiprotein complex at the trans-Golgi network to regulate matrix metalloproteinase secretion. J. Biol. Chem. 291, 462–477. (doi:10.1074/jbc.M115.673582) 26507660 10.1074/jbc.M115.673582 PMC4697185

[8] D'Angelo G *et al.* 2013 Vesicular and non-vesicular transport feed distinct glycosylation pathways in the Golgi. Nature 501, 116–120. (doi:10.1038/nature12423) 23913272 10.1038/nature12423

[9] Funato K, Riezman H 2001 Vesicular and nonvesicular transport of ceramide from ER to the Golgi apparatus in yeast. J. Cell Biol. 155, 949–960. (doi:10.1083/jcb.200105033) 11733544 10.1083/jcb.200105033 PMC2150913

[10] Olayioye MA, Hausser A 2012 Integration of non-vesicular and vesicular transport processes at the Golgi complex by the PKD–CERT network. Biochim. Biophys. Acta (BBA) - Mol. Cell Biol. Lipids 1821, 1096–1103. (doi:10.1016/j.bbalip.2011.12.005) 10.1016/j.bbalip.2011.12.005 22226883

[11] Lev S 2010 Non-vesicular lipid transport by lipid-transfer proteins and beyond. Nat. Rev. Mol. Cell Biol. 11, 739–750. (doi:10.1038/nrm2971) 20823909 10.1038/nrm2971

[12] Hanada K, Kumagai K, Tomishige N, Kawano M 2007 CERT and intracellular trafficking of ceramide. Biochim. Biophys. Acta (BBA) - Mol. Cell Biol. Lipids 1771, 644–653. (doi:10.1016/j.bbalip.2007.01.009) 10.1016/j.bbalip.2007.01.009 17314061

[13] Hanada K, Kumagai K, Tomishige N, Yamaji T 2009 CERT-mediated trafficking of ceramide. Biochim. Biophys. Acta (BBA) - Mol. Cell Biol. Lipids 1791, 684–691. (doi:10.1016/j.bbalip.2009.01.006) 10.1016/j.bbalip.2009.01.006 19416656

[14] Kawano M, Kumagai K, Nishijima M, Hanada K 2006 Efficient trafficking of ceramide from the endoplasmic reticulum to the Golgi apparatus requires a VAMP-associated protein-interacting FFAT motif of CERT. J. Biol. Chem. 281, 30 279–30 288. (doi:10.1074/jbc.M605032200) 10.1074/jbc.M605032200 16895911

[15] De Matteis MA, Rega LR 2015 Endoplasmic reticulum–Golgi complex membrane contact sites. Curr. Opin. Cell Biol. 35, 43–50. (doi:10.1016/j.ceb.2015.04.001) 25950841 10.1016/j.ceb.2015.04.001

[16] Huitema K, van den Dikkenberg J, Brouwers JFHM, Holthuis JCM 2004 Identification of a family of animal sphingomyelin synthases. EMBO J. 23, 33–44. (doi:10.1038/sj.emboj.7600034) 14685263 10.1038/sj.emboj.7600034 PMC1271672

[17] Perry RJ, Ridgway ND 2005 Molecular mechanisms and regulation of ceramide transport. Biochim. Biophys. Acta (BBA) - Mol. Cell Biol. Lipids 1734, 220–234. (doi:10.1016/j.bbalip.2005.04.001) 10.1016/j.bbalip.2005.04.001 15907394

[18] Tafesse FG, Ternes P, Holthuis JCM 2006 The multigenic sphingomyelin synthase family. J. Biol. Chem. 281, 29 421–29 425. (doi:10.1074/jbc.R600021200) 10.1074/jbc.R600021200 16905542

[19] Tafesse FG, Huitema K, Hermansson M, van der Poel S, van den Dikkenberg J, Uphoff A, Somerharju P, Holthuis JCM 2007 Both sphingomyelin synthases SMS1 and SMS2 are required for sphingomyelin homeostasis and growth in human HeLa cells. J. Biol. Chem. 282, 17 537–17 547. (doi:10.1074/jbc.M702423200) 10.1074/jbc.M702423200 17449912

[20] Thomaseth C, Weber P, Hamm T, Kashima K, Radde NE 2013 Modeling sphingomyelin synthase 1 driven reaction at the Golgi apparatus can explain data by inclusion of a positive feedback mechanism. J. Theor. Biol. 9, 174–180. (doi:10.1016/j.jtbi.2013.08.022) 10.1016/j.jtbi.2013.08.022 24001971

[21] Hausser A, Storz P, Märtens S, Link G, Toker A, Pfizenmaier K 2005 Protein kinase D regulates vesicular transport by phosphorylating and activating phosphatidylinositol-4 kinase IIIbeta at the Golgi complex. Nat. Cell Biol. 7, 880–886. (doi:10.1038/ncb1289) 16100512 10.1038/ncb1289 PMC1458033

[22] Hanada K 2006 Discovery of the molecular machinery CERT for endoplasmic reticulum-to-Golgi trafficking of ceramide. Mol. Cell. Biochem. 286, 23–31. (doi:10.1007/s11010-005-9044-z) 16601923 10.1007/s11010-005-9044-z

[23] Levine TP 2007 A lipid transfer protein that transfers lipid. J. Cell Biol. 179, 11–13. (doi:10.1083/jcb.200709055) 17923527 10.1083/jcb.200709055 PMC2064725

[24] Hanada K 2010 Intracellular trafficking of ceramide by ceramide transfer protein. Proc. Jpn. Acad. Ser. B, Phys. Biol. Sci. 86, 426–437. (doi:10.2183/pjab.86.426) 10.2183/pjab.86.426 PMC3417804 20431265

[25] Kumagai K, Kawano M, Shinkai-Ouchi F, Nishijima M, Hanada K 2007 Interorganelle trafficking of ceramide is regulated by phosphorylation-dependent cooperativity between the PH and START domains of CERT. J. Biol. Chem. 282, 17 758–17 766. (doi:10.1074/jbc.M702291200) 10.1074/jbc.M702291200 17442665

[26] Kumagai K, Kawano-Kawada M, Hanada K 2014 Phosphoregulation of the ceramide transport protein CERT at serine 315 in the interaction with VAMP-associated protein (VAP) for inter-organelle trafficking of ceramide in mammalian cells. J. Biol. Chem. 289, 10 748–10 760. (doi:10.1074/jbc.M113.528380) 10.1074/jbc.M113.528380 PMC4036191 24569996

[27] Weber P, Hornjik M, Olayioye MA, Hausser A, Radde NE 2015 A computational model of PKD and CERT interactions at the trans-Golgi network of mammalian cells. BMC Syst. Biol. 9, 9 (doi:10.1186/s12918-015-0147-1) 25889812 10.1186/s12918-015-0147-1 PMC4349302

[28] Florin L, Pegel A, Becker E, Hausser A, Olayioye MA, Kaufmann H 2009 Heterologous expression of the lipid transfer protein CERT increases therapeutic protein productivity of mammalian cells. J. Biotechnol. 141, 84–90. (doi:10.1016/j.jbiotec.2009.02.014) 19428735 10.1016/j.jbiotec.2009.02.014

[29] Santos C *et al.* 2014 Identification of novel CERT ligands as potential ceramide trafficking inhibitors. Chem. BioChem. 15, 2522–2528. (doi:10.1002/cbic.201402366) 10.1002/cbic.201402366 25256104

[30] Fleury L, Faux C, Santos C, Ballereau S, Génisson Y, Ausseil F 2015 Development of a CERT START domain–ceramide HTRF binding assay and application to pharmacological studies and screening. J. Biomol. Screen. 20, 779–787. (doi:10.1177/1087057115573402) 25716975 10.1177/1087057115573402

[31] Rao RP *et al.* 2014 Ceramide transfer protein deficiency compromises organelle function and leads to senescence in primary cells. PLoS ONE 9, e92142 (doi:10.1371/journal.pone.0092142) 24642596 10.1371/journal.pone.0092142 PMC3958450

[32] Alon U 2006 An introduction to systems biology: design principles of biological circuits. London, UK: Chapman & Hall/CRC.

[33] El-Samad H, Prajna S, Papachristodoulou A, Doyle J, Khammash M 2006 Advanced methods and algorithms for biological networks analysis. Proc. IEEE 94, 832–853. (doi:10.1109/JPROC.2006.871776)

[34] Angeli D 2009 A tutorial on chemical reaction network dynamics. Eur. J. Control 15, 398–406. (doi:10.3166/ejc.15.398-406)

[35] Del Vecchio D, Murray RM 2014 Biomolecular feedback systems. Princeton, NJ: Princeton University Press.

[36] Alon U, Surette MG, Barkai N, Leibler S 1999 Robustness in bacterial chemotaxis. Nature 397, 168–171. (doi:10.1038/16483) 9923680 10.1038/16483

[37] Shinar G, Milo R, Martìnez R, Alon U 2007 Input-output robustness in simple bacterial signaling systems. Proc. Natl Acad. Sci. USA 104, 19 931–19 935. (doi:10.1073/pnas.0706792104) 10.1073/pnas.0706792104 PMC2148400 18077424

[38] Kitano H 2004 Biological robustness. Nat. Rev. Genet. 5, 826–837. (doi:10.1038/nrg1471) 15520792 10.1038/nrg1471

[39] Alon U 2007 Network motifs: theory and experimental approaches. Nat. Rev. Genet. 8, 450–461. (doi:10.1038/nrg2102) 17510665 10.1038/nrg2102

[40] Steuer R, Waldherr S, Sourjik V, Kollmann M 2011 Robust signal processing in living cells. PLoS Comput. Biol. 7, e1002218 (doi:10.1371/journal.pcbi.1002218) 22215991 10.1371/journal.pcbi.1002218 PMC3219616

[41] Radde NE, Bar NS, Banaji M 2010 Graphical methods for analysing feedback in biological networks—a survey. Int. J. Syst. Sci. 41, 35–46. (doi:10.1080/00207720903151326)

[42] Shinar G, Feinberg M 2010 Structural sources of robustness in biochemical reaction networks. Science 327, 1389–1391. (doi:10.1126/science.1183372) 20223989 10.1126/science.1183372

[43] Blanchini F, Franco E 2011 Structurally robust biological networks. BMC Syst. Biol. 5, 74 (doi:10.1186/1752-0509-5-74) 21586168 10.1186/1752-0509-5-74 PMC3125314

[44] Giordano G 2016 Structural analysis and control of dynamical networks. PhD dissertation, Università degli Studi di Udine.

[45] Blanchini F, Giordano G 2014 Piecewise–linear Lyapunov functions for structural stability of biochemical networks. Automatica 50, 2482–2493. (doi:10.1016/j.automatica.2014.08.012)

[46] Blanchini F, Giordano G 2017 Polyhedral Lyapunov functions structurally ensure global asymptotic stability of dynamical networks iff the Jacobian is non-singular. Automatica 86, 183–191. (doi:10.1016/j.automatica.2017.08.022)

[47] Al-Radhawi MA, Angeli D 2015 New approach to the stability of chemical reaction networks: piecewise linear in rates Lyapunov functions. IEEE Trans. Autom. Control 61, 76–89. (doi:10.1109/TAC.2015.2427691)

[48] Mincheva M, Craciun G 2008 Multigraph conditions for multistability, oscillations and pattern formation in biochemical reaction networks. Proc. IEEE 96, 1281–1291. (doi:10.1109/JPROC.2008.925474)

[49] Blanchini F, Franco E, Giordano G 2012 Determining the structural properties of a class of biological models. In *Proc. IEEE Conf. on Decision and Control, Maui, HI, USA, December 2012*, pp. 5505–5510. IEEE.

[50] Blanchini F, Franco E, Giordano G 2014 A structural classification of candidate oscillatory and multistationary biochemical systems. Bull. Math. Biol. 76, 2542–2569. (doi:10.1007/s11538-014-0023-y) 25230803 10.1007/s11538-014-0023-y

[51] Blanchini F, Franco E, Giordano G 2015 Structural conditions for oscillations and multistationarity in aggregate monotone systems. In *Proc. IEEE Conf. on Decision and Control, Osaka, Japan, December 2015*, pp. 609–614. IEEE.

[52] Culos GJ, Olesky DD, van den Driessche P 2016 Using sign patterns to detect the possibility of periodicity in biological systems. J. Math. Biol. 72, 1281–1300. (doi:10.1007/s00285-015-0906-z) 26092517 10.1007/s00285-015-0906-z

[53] Giordano G, Cuba Samaniego C, Franco E, Blanchini F 2016 Computing the structural influence matrix for biological systems. J. Math. Biol. 72, 1927–1958. (doi:10.1007/s00285-015-0933-9) 26395779 10.1007/s00285-015-0933-9

[54] Giordano G, Blanchini F 2017 Flow-inducing networks. IEEE Control Syst. Lett. 1, 44–49. (doi:10.1109/LCSYS.2017.2702279)

[55] Baron CL, Malhotra V 2002 Role of diacylglycerol in PKD recruitment to the TGN and protein transport to the plasma membrane. Science 295, 325–328. (doi:10.1126/science.1066759) 11729268 10.1126/science.1066759

[56] Maeda Y, Beznoussenko GV, Lint JV, Mironov AA, Malhotra V 2001 Recruitment of protein kinase D to the trans-Golgi network via the first cysteine-rich domain. EMBO J. 20, 5982–5990. (doi:10.1093/emboj/20.21.5982) 11689438 10.1093/emboj/20.21.5982 PMC125696

[57] Tóth B, Balla A, Ma H, Knight ZA, Shokat KM, Balla T 2006 Phosphatidylinositol 4-kinase III*β* regulates the transport of ceramide between the endoplasmic reticulum and Golgi. J. Biol. Chem. 281, 36 369–36 377. (doi:10.1074/jbc.M604935200) 17003043 10.1074/jbc.M604935200

[58] Rozengurt E, Rey O, Waldron RT 2005 Protein kinase D signaling. J. Biol. Chem. 280, 13 205–13 208. (doi:10.1074/jbc.R500002200) 10.1074/jbc.R500002200 15701647

[59] Wang QJ 2006 PKD at the crossroads of DAG and PKC signaling. Trends Pharmacol. Sci. 27, 317–323. (doi:10.1016/j.tips.2006.04.003) 16678913 10.1016/j.tips.2006.04.003

[60] Fugmann T, Hausser A, Sch'offler P, Schmid S, Pfizenmaier K, Olayioye MA 2007 Regulation of secretory transport by protein kinase D—mediated phosphorylation of the ceramide transfer protein. J. Cell Biol. 178, 15–22. (doi:10.1083/jcb.200612017) 17591919 10.1083/jcb.200612017 PMC2064413

[61] D'Angelo G, Vicinanza M, Campli AD, De Matteis MA 2008 The multiple roles of PtdIns(4)P—not just the precursor of PtdIns(4,5)P2. J. Cell Sci. 121, 1955–1963. (doi:10.1242/jcs.023630) 18525025 10.1242/jcs.023630

[62] Godi A, Pertile P, Meyers R, Marra P, Tullio GD, Iurisci C, Luini A, Corda D, De Matteis MA 1999 ARF mediates recruitment of PtdIns-4-OH kinase-beta and stimulates synthesis of PtdIns(4,5)P2 on the Golgi complex. Nat. Cell Biol. 1, 280–287. (doi:10.1038/12993) 10559940 10.1038/12993

[63] Haynes LP, Sherwood MW, Dolman NJ, Burgoyne RD 2007 Specificity, promiscuity and localization of ARF protein interactions with NCS-1 and phosphatidylinositol-4 kinase- III*β*. Traffic 8, 1080–1092. (doi:10.1111/j.1600-0854.2007.00594.x) 17555535 10.1111/j.1600-0854.2007.00594.x PMC2492389

[64] Lemmon MA, Ferguson KM 2000 Signal-dependent membrane targeting by pleckstrin homology (PH) domains. Biochem. J. 350, 1–18. (doi:10.1042/bj3500001) 10926821 PMC1221219

[65] Levine TP, Munro S 2002 Targeting of Golgi-specific pleckstrin homology domains involves both PtdIns 4-kinase-dependent and -independent components. Curr. Biol. 12, 695–704. (doi:10.1016/S0960-9822(02)00779-0) 12007412 10.1016/s0960-9822(02)00779-0

[66] Alpy F, Tomasetto C 2005 Give lipids a START: the StAR-related lipid transfer (START) domain in mammals. J. Cell Sci. 118, 2791–2801. (doi:10.1242/jcs.02485) 15976441 10.1242/jcs.02485

[67] Kudo N, Kumagai K, Tomishige N, Yamaji T, Wakatsuki S, Nishijima M, Hanada K, Kato R 2008 Structural basis for specific lipid recognition by CERT responsible for nonvesicular trafficking of ceramide. Proc. Natl Acad. Sci. USA 105, 488–493. (doi:10.1073/pnas.0709191105) 18184806 10.1073/pnas.0709191105 PMC2206563

[68] Kumagai K, Yasuda S, Okemoto K, Nishijima M, Kobayashi S, Hanada K 2005 CERT mediates intermembrane transfer of various molecular species of ceramides. J. Biol. Chem. 280, 6488–6495. (doi:10.1074/jbc.M409290200) 15596449 10.1074/jbc.M409290200

[69] Saito S, Matsui H, Kawano M, Kumagai K, Tomishige N, Hanada K, Echigo S, Tamura S, Kobayashi T 2008 Protein phosphatase 2C*ϵ* is an endoplasmic reticulum integral membrane protein that dephosphorylates the ceramide transport protein CERT to enhance its association with organelle membranes. J. Biol. Chem. 283, 6584–6593. (doi:10.1074/jbc.M707691200) 18165232 10.1074/jbc.M707691200

[70] Sontag ED 2014 A technique for determining the signs of sensitivities of steady states in chemical reaction networks. IET Syst. Biol. 8, 251–267. (doi:10.1049/iet-syb.2014.0025) 25478700 10.1049/iet-syb.2014.0025 PMC5653976

[71] Drengstig T, Ueda HR, Ruoff P 2008 Predicting perfect adaptation motifs in reaction kinetic networks. J. Phys. Chem. B 112, 16 752–16 758. (doi:10.1021/jp806818c) 19367864 10.1021/jp806818c

[72] Sontag ED 2003 Adaptation and regulation with signal detection implies internal model. Syst. Control Lett. 50, 119–126. (doi:10.1016/S0167-6911(03)00136-1)

[73] Yi TM, Huang Y, Simon MI, Doyle J 2000 Robust perfect adaptation in bacterial chemotaxis through integral feedback control. Proc. Natl Acad. Sci. USA 97, 4649–4653. (doi:10.1073/pnas.97.9.4649) 10781070 10.1073/pnas.97.9.4649 PMC18287

[74] El-Samad H, Goff JP, Khammash M 2002 Calcium homeostasis and parturient hypocalcemia: an integral feedback perspective. J. Theor. Biol. 214, 17–29. (doi:10.1006/jtbi.2001.2422) 11786029 10.1006/jtbi.2001.2422

[75] El-Samad H, Kurata H, Doyle J, Gross C, Khammash M 2005 Surviving heat shock: Control strategies for robustness and performance. Proc. Natl Acad. Sci. USA 102, 2736–2741. (doi:10.1073/pnas.0403510102) 15668395 10.1073/pnas.0403510102 PMC549435

[76] Ma W, Trusina A, El-Samad H, Lim WA, Tang C 2009 Defining network topologies that can achieve biochemical adaptation. Cell 138, 760–773. (doi:10.1016/j.cell.2009.06.013) 19703401 10.1016/j.cell.2009.06.013 PMC3068210

[77] Shoval O, Goentoro L, Hart Y, Mayo A, Sontag E, Alon U 2010 Fold-change detection and scalar symmetry of sensory input fields. Proc. Natl Acad. Sci. USA 107, 15 995–16 000. (doi:10.1073/pnas.1002352107) 10.1073/pnas.1002352107 PMC2936624 20729472

[78] Mangan S, Alon U 2003 Structure and function of the feed-forward loop network motif. Proc. Natl Acad. Sci. USA 100, 11 980–11 985. (doi:10.1073/pnas.2133841100) 10.1073/pnas.2133841100 PMC218699 14530388

[79] Milo R, Shen-Orr S, Itzkovitz S, Kashtan N, Chklovskii D, Alon U 2002 Network motifs: simple building blocks of complex networks. Science 298, 824–827. (doi:10.1126/science.298.5594.824) 12399590 10.1126/science.298.5594.824

[80] Richeson R, Wiseman J 2002 A fixed point theorem for bounded dynamical systems. Illinois J. Math. 46, 491–495.

[81] Richeson R, Wiseman J 2004 Addendum to: a fixed point theorem for bounded dynamical systems [*Illinois J. Math.* **42**, 491–495 (2002)]. Illinois J. Math. 48, 1079–1080.

[82] Srzednicki R 1985 On rest points of dynamical systems. Fundamenta Mathematicae 126, 69–81. (doi:10.4064/fm-126-1-69-81)

[83] Angeli D, De Lenheer P, Sontag ED 2006 On the structural monotonicity of chemical reaction networks. In *Proc. IEEE Conf. on Decision and Control, San Diego, CA, December 2006*, pp. 7–12. IEEE.

[84] Angeli D, Sontag ED 2004 Interconnections of monotone systems with steady-state characteristics. In *Optimal control, stabilization and nonsmooth analysis* (eds MS de Queiroz, M Malisoff, P Wolenski). Lecture Notes in Control and Information Sciences, vol. 301, pp. 135–154. Berlin, Germany: Springer.

[85] De Lenheer P, Angeli D, Sontag ED 2007 Monotone chemical reaction networks. J. Math. Chem. 41, 295–314. (doi:10.1007/s10910-006-9075-z)

[86] Smith HL 2008 Monotone dynamical systems: an introduction to the theory of competitive and cooperative systems. Providence, RI: American Mathematical Society.

[87] Perry RJ, Ridgway ND 2006 Oxysterol-binding protein and vesicle-associated membrane protein-associated protein are required for sterol-dependent activation of the ceramide transport protein. Mol. Biol. Cell 17, 2604–2616. (doi:10.1091/mbc.e06-01-0060) 16571669 10.1091/mbc.E06-01-0060 PMC1474796

[88] Nhek S, Ngo M, Yang X, Ng MM, Field SJ, Asara JM, Ridgway ND, Toker A 2010 Regulation of oxysterol-binding protein Golgi localization through protein kinase D-mediated phosphorylation. Mol. Biol. Cell 21, 2327–2337. (doi:10.1091/mbc.E10-02-0090) 20444975 10.1091/mbc.E10-02-0090 PMC2893995

[89] Malhotra V, Campelo F 2011 PKD regulates membrane fission to generate TGN to cell surface transport carriers. Cold Spring Harbor Perspect. Biol. 3, a005280 (doi:10.1101/cshperspect.a005280) 10.1101/cshperspect.a005280 PMC3039530 21421913

[90] Holthuis JC, Menon AK 2014 Lipid landscapes and pipelines in membrane homeostasis. Nature 510, 48–57. (doi:10.1038/nature13474) 24899304 10.1038/nature13474

[91] Peretti D, Dahan N, Shimoni E, Hirschberg K, Lev S 2008 Coordinated lipid transfer between the endoplasmic reticulum and the Golgi complex requires the VAP proteins and is essential for Golgi-mediated transport. Mol. Biol. Cell 19, 3871–3884. (doi:10.1091/mbc.e08-05-0498) 18614794 10.1091/mbc.E08-05-0498 PMC2526681

[92] Strating JR *et al.* 2015 Itraconazole inhibits enterovirus replication by targeting the oxysterol-binding protein. Cell Rep. 10, 600–615. (doi:10.1016/j.celrep.2014.12.054) 25640182 10.1016/j.celrep.2014.12.054 PMC4383725

[93] Mesmin B, Bigay J, Moser von Filseck J, Lacas-Gervais S, Drin G, Antonny B 2013 A four-step cycle driven by PI(4)P hydrolysis directs sterol/PI(4)P exchange by the ER-Golgi tether OSBP. Cell 155, 830–843. (doi:10.1016/j.cell.2013.09.056) 24209621 10.1016/j.cell.2013.09.056

[94] Mesmin B, Antonny B 2016 The counterflow transport of sterols and PI4P. Biochim. Biophys. Acta 1861, 940–951. (doi:10.1016/j.bbalip.2016.02.024) 26928592 10.1016/j.bbalip.2016.02.024

[95] Capasso S *et al.* 2017 Sphingolipid metabolic flow controls phosphoinositide turnover at the *trans*-Golgi network. EMBO J. 36, 1736–1754. (doi:10.15252/embj.201696048) 28495678 10.15252/embj.201696048 PMC5470045

[96] Alam-Nazki A, Krishnan J 2015 Spatial control of biochemical modification cascades and pathways. Biophys. J. 108, 2912–2924. (doi:10.1016/j.bpj.2015.05.012) 26083931 10.1016/j.bpj.2015.05.012 PMC4472220

[97] Menon G, Okeke C, Krishnan J 2017 Modelling compartmentalization towards elucidation and engineering of spatial organization in biochemical pathways. Sci. Rep. 7, 12057 (doi:10.1038/s41598-017-11081-8) 28935941 10.1038/s41598-017-11081-8 PMC5608717

[98] Cuba Samaniego C, Giordano G, Kim J, Blanchini F, Franco E 2016 Molecular titration promotes oscillations and bistability in minimal network models with monomeric regulators. ACS Synth. Biol. 5, 321–333. (doi:10.1021/acssynbio.5b00176) 26797494 10.1021/acssynbio.5b00176

[99] Cuba Samaniego C, Giordano G, Blanchini F, Franco E 2017 Stability analysis of an artificial biomolecular oscillator with non-cooperative regulatory interactions. J. Biol. Dyn. 11, 102–120. (doi:10.1080/17513758.2016.1245790) 10.1080/17513758.2016.1245790 27830588

