# Supplementary material for: Ceramide-transfer protein-mediated ceramide transfer is a structurally tunable flow-inducing mechanism with structural feed-forward loops
Source: R Soc Open Sci. 2018 Jun 27;5(6):180494. doi: 10.1098/rsos.180494 (PMC6030332; doi:10.1098/rsos.180494)
Supplement: Supplementary material [file rsos180494supp1.pdf]

CERT-mediated ceramide transfer is a structurally tunable flow-inducing mechanism with structural feed-forward loops

—

Supplementary Material

—

Giulia Giordano[1]

[1]*Delft Center for Systems and Control, Delft University of Technology, Mekelweg 2, 2628 CD Delft, The Netherlands.* g.giordano@tudelft.nl

Contents

1 Systems and Jacobians **3**

2 Structural Steady-State Input-Output Influences **4**

3 Flow-Inducing Networks: System Reformulation **6**

4 Structural Flow-Inducing Mechanisms **7**

1 Systems and Jacobians

The two mathematical descriptions of the models are reported here for simplicity. The **short distance shuttle** system is

$$\begin{cases} \dot{x}_1 = -x_1 h(x_4, x_5) - x_1 f_1(u_1) + x_2 f_2(u_2) + s_1 - \alpha x_1 \\ \dot{x}_2 = x_1 h(x_4, x_5) + x_1 f_1(u_1) - x_2 f_2(u_2) - \beta x_2 \end{cases}$$
$$\begin{cases} \dot{x}_3 = \kappa x_4 - x_3 g_1(x_2) + s_3 - \gamma x_3 \\ \dot{x}_4 = -\kappa x_4 + x_3 g_1(x_2) - \delta x_4 \end{cases} \quad (1)$$
$$\begin{cases} \dot{x}_5 = \lambda x_6 - x_5 g_4(x_4) + s_5 - \varepsilon x_5 \\ \dot{x}_6 = -\lambda x_6 + x_7 g_2(x_2) - \zeta x_6 \\ \dot{x}_7 = x_5 g_4(x_4) - x_7 g_2(x_2) - \eta x_7 \end{cases}$$

while the **neck swinging** system is

$$\begin{cases} \dot{x}_1 = -x_1 g_7(x_7) - x_1 f_1(u_1) + x_2 f_2(u_2) + s_1 - \alpha x_1 \\ \dot{x}_2 = x_1 g_7(x_7) + x_1 f_1(u_1) - x_2 f_2(u_2) - \beta x_2 \end{cases}$$
$$\begin{cases} \dot{x}_3 = \kappa x_4 - x_3 g_1(x_2) + s_3 - \gamma x_3 \\ \dot{x}_4 = -\kappa x_4 + x_3 g_1(x_2) - \delta x_4 \end{cases} \quad (2)$$
$$\begin{cases} \dot{x}_5 = -\nu x_5 + \lambda x_6 + s_5 - \varepsilon x_5 \\ \dot{x}_6 = \nu x_5 - \lambda x_6 - x_6 g_4(x_4) + x_7 g_2(x_2) - \zeta x_6 \\ \dot{x}_7 = x_6 g_4(x_4) - x_7 g_2(x_2) - \eta x_7 \end{cases}$$

The Jacobian matrix corresponding to system (1) is

$$J_{SDS} = \begin{bmatrix} -(\alpha+\vartheta+\xi) & \pi & 0 & -\nu & -\mu & 0 & 0 \\ \vartheta+\xi & -(\beta+\pi) & 0 & \nu & \mu & 0 & 0 \\ 0 & -\rho & -(\gamma+\sigma) & \kappa & 0 & 0 & 0 \\ 0 & \rho & \sigma & -(\delta+\kappa) & 0 & 0 & 0 \\ 0 & 0 & 0 & -\psi & -(\varepsilon+\varphi) & \lambda & 0 \\ 0 & \tau & 0 & 0 & 0 & -(\zeta+\lambda) & \omega \\ 0 & -\tau & 0 & \psi & \varphi & 0 & -(\eta+\omega) \end{bmatrix} \quad (3)$$

where $\vartheta = h(\bar{x}_4, \bar{x}_5)$, $\mu = \bar{x}_1\, \partial h(x_4, x_5)/\partial x_5|_{(\bar{x}_4, \bar{x}_5)}$, $\nu = \bar{x}_1\, \partial h(x_4, x_5)/\partial x_4|_{(\bar{x}_4, \bar{x}_5)}$, $\xi = f_1(\bar{u}_1)$, $\pi = f_2(\bar{u}_2)$, $\rho = \bar{x}_3\, \partial g_1(x_2)/\partial x_2|_{\bar{x}_2}$, $\sigma = g_1(\bar{x}_2)$, $\tau = \bar{x}_7\, \partial g_2(x_2)/\partial x_2|_{\bar{x}_2}$, $\omega = g_2(\bar{x}_2)$, $\psi = \bar{x}_5\, \partial g_4(x_4)/\partial x_4|_{\bar{x}_4}$, $\varphi = g_4(\bar{x}_4)$.

The Jacobian matrix for system (2) is

$$J_{NS} = \begin{bmatrix} -(\alpha+\vartheta+\xi) & \pi & 0 & 0 & 0 & 0 & -\mu \\ \vartheta+\xi & -(\beta+\pi) & 0 & 0 & 0 & 0 & \mu \\ 0 & -\rho & -(\gamma+\sigma) & \kappa & 0 & 0 & 0 \\ 0 & \rho & \sigma & -(\delta+\kappa) & 0 & 0 & 0 \\ 0 & 0 & 0 & 0 & -(\varepsilon+\nu) & \lambda & 0 \\ 0 & \tau & 0 & -\psi & \nu & -(\zeta+\lambda+\varphi) & \omega \\ 0 & -\tau & 0 & \psi & 0 & \varphi & -(\eta+\omega) \end{bmatrix} \quad (4)$$

where $\vartheta = g_7(\bar{x}_7)$, $\mu = \bar{x}_1\, \partial g_7(x_7)/\partial x_7|_{\bar{x}_7}$, $\xi = f_1(\bar{u}_1)$, $\pi = f_2(\bar{u}_2)$, $\rho = \bar{x}_3\, \partial g_1(x_2)/\partial x_2|_{\bar{x}_2}$, $\sigma = g_1(\bar{x}_2)$, $\tau = \bar{x}_7\, \partial g_2(x_2)/\partial x_2|_{\bar{x}_2}$, $\omega = g_2(\bar{x}_2)$, $\psi = \bar{x}_6\, \partial g_4(x_4)/\partial x_4|_{\bar{x}_4}$, $\varphi = g_4(\bar{x}_4)$.

2 Structural Steady-State Input-Output Influences

Given a generic nonlinear system, a certain variable of the system can be seen as the system *output* and another relevant variable or parameter of the system as the system *input*. Then, the *steady-state input-output influence* is the ensuing variation of the steady state of a certain variable of the system (seen as the system output), upon a variation in a relevant variable or parameter (which can be seen as an input for the system). Of course, different variables of the system may respond with a steady-state variation that has the same sign as the input variation, the opposite sign, or is zero. The steady-state input-output influence is *structurally signed* if it always has the same sign (positive, negative, or zero), regardless of the choice of parameter values [5].

To assess the steady-state input-output influence in the system

$$\dot{x}(t) = f(x(t), u(t)), \quad (5)$$
$$y(t) = g(x(t)), \quad (6)$$

where f and g are continuously differentiable, $x \in \mathbb{R}^n$, u is a scalar input and y a scalar output, assume that there exists an asymptotically stable equilibrium point $\bar{x}$, corresponding to $\bar{u}$, such that $f(\bar{x}, \bar{u}) = 0$. Then, both the state asymptotic value $\bar{x}(u)$ and the output asymptotic value $\bar{y}(u) = g(\bar{x})$ are functions of u. If the considered input variation is small enough to ensure that asymptotic stability of $\bar{x}(u)$ is preserved (being the eigenvalues of the Jacobian matrix continuously dependent on the entries, which are in turn continuous functions of u), then the implicit function theorem provides an analytic expression for the derivative of the steady-state input-output map that relates y to u in system (5)–(6):

$$\frac{\partial \bar{y}}{\partial \bar{u}} = \left.\frac{\partial g}{\partial x}\right|_{\bar{x}} \left(- \left.\frac{\partial f}{\partial x}\right|_{(\bar{x},\bar{u})} \right)^{-1} \left.\frac{\partial f}{\partial u}\right|_{(\bar{x},\bar{u})}.$$

Consider the linear approximation of the nonlinear system in a neighborhood of the equilibrium $\bar{x}$, with $z(t) = x(t) - \bar{x}$, $v(t) = u(t) - \bar{u}$, $w(t) = y(t) - \bar{y}$:

$$\dot{z}(t) = Jz(t) + Ev(t),$$
$$w(t) = Hz(t),$$

where $J_{ij} = \left.\frac{\partial f_i}{\partial x_j}\right|_{(\bar{x},\bar{u})}$, $E_i = \left.\frac{\partial f_i}{\partial u}\right|_{(\bar{x},\bar{u})}$ and $H_i = \left.\frac{\partial g}{\partial x_i}\right|_{\bar{x}}$: J is the Jacobian matrix computed at the equilibrium, while E and H are a column and a row vector that represent, respectively, how the input acts on the system state and how the output depends on the system state in the linearised system.

Then, following [5], the input-output influence can be expressed as

$$\frac{\partial \bar{y}}{\partial \bar{u}} = H(-J)^{-1}E = \frac{n(0)}{d(0)},$$

where $d(0) = \det(-J) > 0$ if the Jacobian is computed at a stable equilibrium, while

$$n(0) = \det \begin{bmatrix} -J & -E \\ H & 0 \end{bmatrix}. \quad (7)$$

The above expression can be used to evaluate input-output influences for a given choice of parameters. However, it is particularly interesting to assess the structural (parameter-free) input-output influence,

namely to check if, upon a perturbation due to a constant input, *for any feasible choice of the model parameters*, the ensuing variation of the steady-state input value has the same sign of the influence, the opposite sign, or is zero (and in this case the influence is structurally signed), or if the sign of the variation depends on the choice of parameter values. To structurally evaluate the sign of steady-state input-output influences, the vertex algorithm proposed in [5] can be applied if the system admits the so-called BDC-decomposition [1, 2, 3, 5], namely, its Jacobian can be written in the form $J = \sum_{i=1}^{q} d_i M_i$, where the M_i's are rank-one matrices and the d_i's are positive scalars related to the system partial derivatives (see [3, 5] for details).

Systems (1) and (2) do admit a BDC-decomposition, where the d_i's correspond to the Greek letters in the expressions (3) and (4). Hence, the vertex algorithm can be applied to structurally evaluate the sign of steady-state input-output influences in these systems.

The *influence matrix*, whose (i, j) entry expresses the sign of the overall steady-state influence on the ith system variable of an external persistent additive input applied to the dynamic equation of the jth system variable, can be evaluated as follows. As shown in [5], given a system of the form

$$\dot{x}(t) = f(x(t)) + Eu(t), \tag{8}$$

$$y(t) = Hx(t), \tag{9}$$

with Jacobian

$$J = \left.\frac{\partial f(x)}{\partial x}\right|_{x=\bar{x}},$$

where $\bar{x}$ is an asymptotically stable equilibrium, it is enough to take vectors $E = E_j$ and $H = H_i$ with a single non-zero entry equal to one

$$E_j = [0 \ \dots \ 0 \ \underbrace{1}_{\text{position } j} \ 0 \ \dots \ 0]^\top, \qquad H_i = [0 \ \dots \ 0 \ \underbrace{1}_{\text{position } i} \ 0 \ \dots \ 0].$$

Then, if the system admits a BDC-decomposition, the vertex algorithm in [5] can be used to evaluate each entry $[\Sigma]_{ij}$ of the *structural influence matrix* $\Sigma \in \mathbb{R}^{n \times n}$, thus obtaining:

- '$+1$' if the influence is positive for any feasible choice of the parameters;
- '0' if there is perfect adaptation for any feasible choice of the parameters;
- '-1' if the influence is negative for any feasible choice of the parameters;
- '$?$' if the influence can have a different sign depending on the chosen parameters.

As mentioned earlier, the determinant $\det(-J)$ must be positive if the equilibrium is stable. If the system is structurally stable, with $\det(-J) > 0$ for any possible value of the parameters, the signs provided by the computation of the steady-state influence matrix, or of any steady-state input-output influence, are valid no matter how the parameters are chosen. However, even when $\det(-J)$ is not sign determined, the influences can still be computed based on the vertex algorithm, with the caution that the provided outcome will be valid only around *stable* equilibrium points. This is the case for the considered two models, since $\det(J_{SDS})$ and $\det(J_{NS})$ are indeed not sign determined.

3 Flow-Inducing Networks: System Reformulation

Formally, the mathematical framework in [4] requires that all the interconnected subsystems are *compartmental*, namely, subject to mass conservation. This becomes true for both system (1) and system (2) if the external inflows s_i and the self-degradation terms for each species are neglected: this amounts to assuming that an external production rate compensates degradation, which is a standard assumption in the analysis of biomolecular systems. Under this assumption, the short distance shuttle system becomes

$$\begin{cases} \dot{x}_1 = -x_1 h(x_4, x_5) - x_1 f_1(u_1) + x_2 f_2(u_2) \\ \dot{x}_2 = x_1 h(x_4, x_5) + x_1 f_1(u_1) - x_2 f_2(u_2) \end{cases} \\ \begin{cases} \dot{x}_3 = \kappa x_4 - x_3 g_1(x_2) \\ \dot{x}_4 = -\kappa x_4 + x_3 g_1(x_2) \end{cases} \\ \begin{cases} \dot{x}_5 = \lambda x_6 - x_5 g_4(x_4) \\ \dot{x}_6 = -\lambda x_6 + x_7 g_2(x_2) \\ \dot{x}_7 = x_5 g_4(x_4) - x_7 g_2(x_2) \end{cases} \tag{10}$$

and the neck swinging system becomes

$$\begin{cases} \dot{x}_1 = -x_1 g_7(x_7) - x_1 f_1(u_1) + x_2 f_2(u_2) \\ \dot{x}_2 = x_1 g_7(x_7) + x_1 f_1(u_1) - x_2 f_2(u_2) \end{cases} \\ \begin{cases} \dot{x}_3 = \kappa x_4 - x_3 g_1(x_2) \\ \dot{x}_4 = -\kappa x_4 + x_3 g_1(x_2) \end{cases} \\ \begin{cases} \dot{x}_5 = -\nu x_5 + \lambda x_6 \\ \dot{x}_6 = \nu x_5 - \lambda x_6 - x_6 g_4(x_4) + x_7 g_2(x_2) \\ \dot{x}_7 = x_6 g_4(x_4) - x_7 g_2(x_2) \end{cases} \tag{11}$$

It is now apparent that the total concentrations of PKD, PI4KIIIβ and CERT remain constant, hence in each subsystem there is total mass conservation: $x_1 + x_2 = \text{PKD}^{tot}$, $x_3 + x_4 = \text{PI4KIII}\beta^{tot}$ and $x_5 + x_6 + x_7 = \text{CERT}^{tot}$. Although just systems (10) and (11) exactly fit in the mathematical framework proposed in [4], since they are composed of compartmental systems, the very same flow-inducing effects can be identified in the original systems (1) and (2). Hence, just the original systems have been studied for the purpose of the present work.

4 Structural Flow-Inducing Mechanisms

This section provides more details on the computation of structurally signed influences that reveal the structural flow-inducing mechanism in the two models.

In the short distance shuttle system, if Φ_{SDS} is chosen as the system output, then the structural influence on this output can be computed from (7) by setting

$$H = \begin{bmatrix} 0 & \tau & 0 & \psi & \varphi & \lambda & \omega \end{bmatrix}.$$

In the neck swinging system, if Φ_{NS} is chosen as the system output and a persistent input is applied to the equation of x_2 (concentration of active PKD), then the structural influences can be computed by plugging into (7) an output matrix

$$H = \begin{bmatrix} 0 & \tau & 0 & \psi & 0 & \varphi & \omega \end{bmatrix}.$$

In both models, if the persistent input is u_1, then the input matrix in (7) is

$$E = \begin{bmatrix} -\varsigma & \varsigma & 0 & 0 & 0 & 0 & 0 \end{bmatrix}^\top,$$

where $\varsigma = \bar{x}_1 \partial f_1(u_1)/\partial u_1|_{\bar{u}_1}$. Conversely, if a persistent input is applied, for instance, to the equation of x_2 (concentration of active PKD), then

$$E = \begin{bmatrix} 0 & 1 & 0 & 0 & 0 & 0 & 0 \end{bmatrix}^\top.$$

References

[1] Blanchini F, Franco E, Giordano G. 2012. Determining the structural properties of a class of biological models. *Proceedings of the IEEE Conference on Decision and Control*, pp. 5505–5510.

[2] Blanchini F, Giordano G. 2014. Piecewise–linear Lyapunov functions for structural stability of biochemical networks. *Automatica*, 50(10), pp. 2482–2493.

[3] Giordano G. 2016. Structural analysis and control of dynamical networks. Ph.D. dissertation, Università degli Studi di Udine.

[4] Giordano G, Blanchini F. 2017. Flow-inducing networks. *IEEE Control Systems Letters*, 1(1), pp. 44-49.

[5] Giordano G, Cuba Samaniego C, Franco E, Blanchini F. 2016. Computing the structural influence matrix for biological systems. *Journal of Mathematical Biology*, 72 (7), pp. 1927–1958.

[6] Weber P, Hornjik M, Olayioye MA, Hausser A, Radde NE. 2015. A computational model of PKD and CERT interactions at the trans-Golgi network of mammalian cells. *BMC Systems Biology*, 9(9).
